# Deafness due to loss of a TRPV channel eliminates mating behavior in *Aedes aegypti* males

**DOI:** 10.1073/pnas.2404324121

**Published:** 2024-11-04

**Authors:** Yijin Wang, Dhananjay Thakur, Emma Duge, Caroline Murphy, Ivan Girling, Nicolas A. DeBeaubien, Jieyan Chen, Benjamin H. Nguyen, Adishthi S. Gurav, Craig Montell

**Affiliations:** ^a^Department of Molecular, Cellular, and Developmental Biology, and the Neuroscience Research Institute, University of California, Santa Barbara, CA 93106

**Keywords:** *Aedes aegypti*, TRP channels, hearing, rapid frequency modulation, mating

## Abstract

Attraction and mating between male and female animals depend on effective communication between conspecifics. However, in mosquitoes, we have only a rudimentary understanding of the sensory cues and receptors critical for the communication that is essential for reproductive behavior. While it is known that male *Aedes aegypti* use sound to help them identify females, it is not unclear whether sound detection is absolutely required since other cues such as vision may also participate in mating behavior. To determine the effect of eliminating hearing on mating success, we knocked out the *Ae. aegypti* TRPVa channel, which is a protein expressed in chordotonal neurons in the Johnston’s organ (JO) that respond to sound-induced movements in the antenna. Loss of *trpVa* eradicated sound-induced responses from the JO, thereby abolishing hearing. Strikingly, mutation of *trpVa* eliminated mating behavior in males. In contrast, *trpVa*-null females mated, although this behavior was slightly delayed relative to wild-type females. Males and females produce sounds as they beat their wings at distinct frequencies during flight. Sound mimicking the female wingbeat induced flight, attraction, and copulatory-like behavior in wild-type males without females present, but not in *trpVa*-null males. Males are known to modulate their wingbeat frequencies before mating in the air, which is a phenomenon referred to as rapid frequency modulation (RFM). We found that RFM was absent in mosquitoes lacking TRPVa. We conclude that the requirement for *trpVa* and hearing for male reproductive behavior in *Aedes* is absolute, as mating in the deaf males is eliminated.

## Significance

The modes of communication required for mating in mosquitoes that transmit pathogens causing malaria, dengue, Zika, and other diseases are poorly understood. We addressed this question in *Aedes aegypti,* which spreads viruses infecting ~400 million people annually. It is established that *Aedes* males are attracted to the female wingbeat. However, it was not known whether loss of hearing would just compromise or eradicate mating. We created deaf mosquitoes by eliminating the Transient Receptor Potential Va (TRPVa) channel—a protein required for sound-induced activation of auditory neurons. We found that mating was abolished in deaf males, demonstrating that hearing and TRPVa are essential for male mating behavior. This work reveals a mode of communication that is strictly required for male mating success in a mosquito disease vector.

Sexual attraction and mating typically depend on multiple senses, including olfaction, vision, touch, taste, and hearing. Many animals use redundant sensory pathways to coordinate mating, so that loss or lack of stimulation of one sense does not prevent successful reproduction. The fruit fly, *Drosophila melanogaster*, exemplifies this phenomenon. Courtship behavior in fruit flies is multimodal and begins with a male sensing and approaching a potential mate through detecting volatile pheromones and visual stimuli (1–3). Male fruit flies then vibrate a wing to generate a courtship song to stimulate female receptivity. Male and female fruit flies also communicate with each other through volatile and nonvolatile pheromones, as well as visual and tactile cues (1–3). However, fruit flies that are blind, deaf, or cannot sense pheromones can still copulate and reproduce (4, 5). Nevertheless, the salience of different senses for sexual attraction varies within the animal kingdom.

In contrast to fruit flies, which mate on the ground for many minutes, mosquitoes such as *Aedes* (*Ae.*) *aegypti* copulate in the air. This aerial mating lasts between a few seconds to just under a minute (6, 7). While reproductive behavior in female mosquitoes has been examined in some detail (8–13), less is known regarding male mating behavior. Unraveling the behavioral, cellular, and molecular mechanisms underlying *Ae. aegypti* reproduction is of great interest since these mosquitoes spread viruses that cause dengue, yellow fever, Zika, and other diseases. ~400 million are infected with the dengue virus each year, leading to ~100 million with dengue disease (14). A major concern is that the incidence of dengue is on the rise (15–18).

It has long been known that mosquito reproductive behavior involves communication through the sense of hearing and may represent a mating call (7, 19, 20). Both males and females produce wing vibrations at different frequencies (7, 21, 22). Additionally, the sound that females produce is attractive to males (7), and the mosquitoes modify their wingbeat frequencies (WBFs) at close range in a phenomenon referred to as rapid frequency modulation (RFM) (23). Since other cues such as vision may also promote mating, we wondered whether deafness would eliminate mating behavior by *Ae. aegypti* or whether the impact would be less severe as in *Drosophila*.

To establish whether loss of audition would impair or abolish successful reproductive behavior in *Ae. aegypti*, we attempted to produce deaf mosquitoes using CRISPR-Cas9. To do so, we took advantage of the observation that in another dipteran, *D. melanogaster*, several TRP channels are required for auditory reception, one of which is Iav (24–29). Therefore, we disrupted the gene encoding the Iav homolog in *Ae. aegypti*, TRPVa, and tested whether the *Aedes* mutants were responsive to sounds. We created three mutant alleles (*trpVa^1^*, *trpVa^2^*, and *trpVa^QF2^*), one of which included an in-frame insertion of *QF2* (*trpVa^QF2^*), which drove reporter expression in auditory neurons. The mosquito’s auditory neurons are in the Johnston’s organs (JO), which are located at the base of the antennae (7, 20, 29, 30). We performed sound-evoked field recordings from the JO and found that the transheterozygous mutant *trpVa^1/2^* males and females were unresponsive to sounds and were therefore deaf. The *trpVa^1/2^* mutant females mated, although the time needed to execute this behavior increased. Strikingly, loss of auditory communication eradicated reproductive behavior in *trpVa^1/2^* mutant males, while the heterozygous males (*trpVa^1/+^* and *trpVa^2/+^*) mated as effectively wild-type males. Therefore, auditory stimulation is absolutely required for male mating success, but not for female *Ae. aegypti*.

## Results

### Creation of Deaf *Ae. aegypti*.

**Fig. 1.**
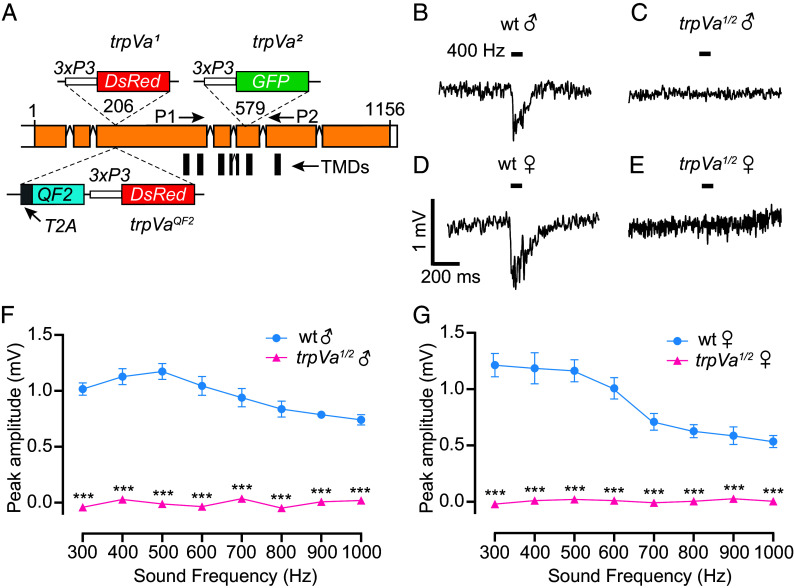
*trpVa* is required for sound-evoked potentials. (*A*) Diagram of the *trpVa* gene and the insertion sites used to create the *trpVa^1^*, *trpVa^2^*, and *trpVa^QF2^* alleles. The numbers correspond to the encoded amino acids. The vertical bars represent the six TMDs. The 4th TMD is encoded by two exons. P1 and P2 indicate the primers used to perform the RT-PCR in wild-type and *trpVa^2^*. (*B–E*) Traces showing sound-evoked potentials in the JO in response to 400 Hz sound stimulation. The stimuli were 50 ms (black bars). (*B*) Wild-type male. (*C*) *trpVa^1/2^* male. (*D*) Wild-type female. (*E*) *trpVa^1/2^* female. (*F* and *G*) Average amplitudes (mV) induced in the JO by sound stimuli at different frequencies. (*F*) Males. (*G*) Females. n = 5 to 8. Means ± SEMs. Two-way ANOVA with Sidak’s multiple comparison for *F* and *G*. ***P < 0.001.

To determine the impact of deafness on mating success in *Ae. aegypti*, we set out to generate mosquitoes that were unable to hear sound stimuli using CRISPR-Cas9. To create a mutation that might cause deafness, we focused on the *trpVa* gene (*AAEL020482*). This gene encodes a protein that is 74.8% identical to the *Drosophila* TRPV channel Iav (*SI Appendix*, Figs. S1 and S2), which fruit flies require for the initial detection of sound (24). We initially created two alleles—one containing *3xP3-DsRed* (*trpVa^1^*) and the other *3xP3-GFP* (*trpVa^2^*). We differentiated transheterozygous mutants from heterozygous mosquitoes by screening for insects that contained both markers. The insertions interrupted either the N-terminal-coding region (*trpVa^1^*) or the coding region for the fifth transmembrane domain (TMDs) (*trpVa^2^*) (Fig. 1*A* and *SI Appendix*, Fig. S2).

To assess whether loss of *trpVa* eliminates auditory reception, we recorded extracellular potentials in response to near-field sound stimuli from the JO (20). The JO, which is located in the pedicel at the base of the antennae, is a collection of mechanosensory neurons including those that respond to sound-induced vibrations of the flagellum (7, 28–30). Sound vibrations activate ciliated bipolar neurons comprised in the many repeating chordotonal units of the JO (28, 29). The JO of male mosquitoes is the largest chordotonal organ known in insects, containing ~16,000 neurons (31). Female mosquitoes possess about half this number.

To determine whether loss of *trpVa* reduced or eliminated the sensation of sound-induced vibrations by the JO, we produced 300 to 1,000 Hz sine tones for 50 ms using a speaker that delivered 83 dB at the position of the mosquito. Similar to previous studies (21, 32), the JO of wild-type males and females responded to sounds at all frequencies tested in the 300 to 1,000 Hz range (Fig. 1 *B*, *D*, *F*, and *G* and *SI Appendix*, Fig. S3 *A*, *C*, *E*, and *F*). Males showed a maximum field potential response at 500 Hz (Fig. 1*F* and *SI Appendix*, Fig. S3*E*). Females responded with the highest amplitudes between 300 and 500 Hz (Fig. 1*G* and *SI Appendix*, Fig. S3*F*). To test for possible movement artifacts from the electrode that could impact the JO recordings, we recorded from dead wild-type mosquitoes and observed no measurable responses to sound stimuli (*SI Appendix*, Fig. S3*G*). To test whether any movements from a live mosquito generated mechanical artifacts in response to the applied sound, we placed the recording electrode on the eye since this organ does not typically respond to auditory stimuli. We did not detect any significant responses to sound from the eye (*SI Appendix*, Fig. S3*G*).

In contrast to wild-type mosquitoes, antennal sound-evoked potentials were eliminated in the *trpVa^1/2^* males and females (Fig. 1 *C* and *E*–*G* and *SI Appendix*, Fig. S3 *B* and *D–F*). These data demonstrate that *trpVa^1/2^* mutants are deaf, and this deficit is due to the failure to activate mechanosensory responses in the JO.

### TRPVa Is Expressed in Auditory Neurons with Sexual Dimorphic Innervation.

**Fig. 2.**
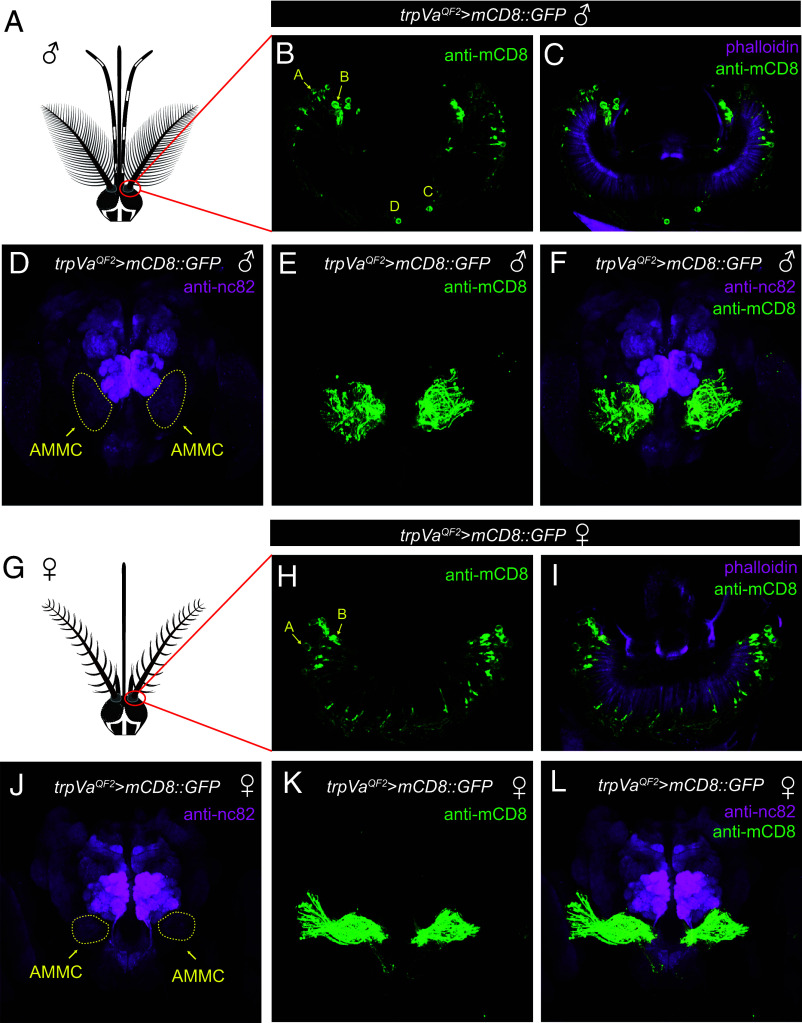
*trpVa* is expressed in the JO with sexually dimorphic axonal projections. (*A*) Cartoon of a male head. (*B* and *C*) Expression of the *trpVa* reporter (*trpVa^QF2^*>*QUAS-mCD8-GFP*) in a cross-section of a JO from a male. The mCD8-GFP (*trpVa* reporter) was detected using anti-mCD8 (green) and F-actin was labeled using a fluorescently labeled phalloidin (magenta). Regions with type A–D scolopidia are indicated. (*D*–*F*) Axonal projections labeled with the *trpVa* reporter in a male brain. Anti-nc82 (magenta) broadly labels neuropils throughout the brain (40). (*G*) Cartoon of a female head. (*H* and *I*) Expression of the *trpVa* reporter in a cross-section of a JO from a female. (*J*–*L*) Axonal projections labeled with the *trpVa* reporter in a female brain.

To determine whether *trpVa* is expressed in JO chordotonal neurons, we generated an in-frame insertion of the *QF2* transcriptional activator gene (33, 34) into the *trpVa* locus using the same guide RNA that we used to create *trpVa^1^* (Fig. 1*A*; *trpVa^QF2^*). Additionally, we included a T2A self-cleaving peptide linker upstream of QF2 so that the QF2 protein would separate from TRPVa (Fig. 1*A*). We also created a *QUAS-mCD8::GFP* reporter line to detect expression of the QF2 driver. The *mCD8:GFP* reporter labeled a subset of the chordotonal neurons in the JO of both males and females (Fig. 2 *A*–*C* and *G*–*I*). In *Ae. aegypti* males, JO neurons are divided into four classes, A–D, while females appear to contain just three classes (A–C) (31, 35). The precise roles of the various classes have not been clarified in *Aedes*, although in *Drosophila* the A and B neurons detect sound, while the class C detect wind and gravity (27–29, 36–39). In males, the staining was concentrated at the highest density in the apical region at the anterior part of the JO in the region previously shown to contain class A and B JO neurons (Fig. 2 *B* and *C*). In addition, we also detected a pair of neurons at the proximal end, which may correspond to the class C and D JO neurons (Fig. 2 *B* and *C*). In females, we detected expression in A and B neurons, but not in C neurons (Fig. 2 *H* and *I*). The axons of the *trpVa^+^* neurons project to the antennal mechanosensory and motor center (AMMC) region in the brain (Fig. 2 *D*–*F* and *J*–*L*). We found that the axonal projections of *trpVa^+^* neurons in the AMMC are sexually dimorphic and are more dispersed in males than in females (Fig. 2 *E*, *F*, *K*, and *L*).

### Deaf *trpVa* Males Fail to Fertilize Females.

Hearing is involved in initiating mating behavior by male *Ae. aegypti* (7, 19, 20). However, given the possibility that other cues, such as vision, also participate in mating, the impact of eliminating hearing on mating is not known. The generation of *trpVa* mutants allows us to study the role of sound as an isolated variable for mating. On average, *Ae. aegypti* complete copulation and insemination within ~20 s of a male contacting a female, and almost always in less than a minute (7). Copulatory behavior is associated with looping or zig-zag flight patterns, and this behavior can occur in small aggregations or even with a single male in flight (41–43).

**Fig. 3.**
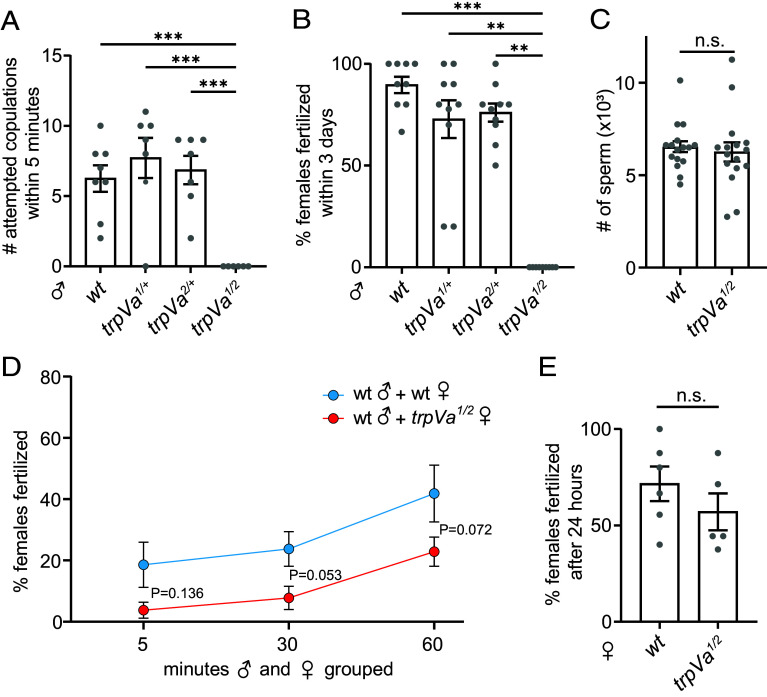
Mutation of *trpVa* eliminates male mating behavior and delays female mating. (*A*) Number of attempted copulations by single male mosquitoes exposed to 10 wild-type virgin females for 5 min (n = 6-8). (*B*) Percentages of fertilized females. 9-11 wild-type females were grouped with 5 males for 3 d (n = 9-10 cages). (*C*) Number of sperm in individual wild-type and *trpVa^1/2^* males. (*D*) Percentages of females that produced progeny when 9-11 virgin females were grouped with one wild-type male for 5, 30, and 60 min (n = 7-8 trials). The differences between wild-type and *trpVa^1/2^* were not significant. (*E*) Percentages of fertilized females when 7-11 virgin females were grouped with 3 wild-type males for 24 h (n = 5-6 trials). *P* = 0.355. Kruskal–Wallis test with Dunn’s multiple comparison test for *A* and *B*. Mann–Whitney *U* test for *C* and *E*. Two-way ANOVA with Sidak’s multiple comparisons test for *D*. Means ± SEMs. n.s., not significant. **P < 0.01, and ***P < 0.001.

To perform a short-term mating assay, we grouped males with females in a 15 × 15 × 15 cm cage for 5 min. Despite the small size of these cages, we consistently observed looping or zig-zag flight patterns with wild-type male mosquitoes (Movie S1), which are associated with in-flight mating (Movie S2) (42). To examine sexual behavior exhibited by the *trpVa* males, we inserted one male and 10 females in each cage, and scored attempted copulations by counting the number of times the male made physical contact with a female. Wild-type and heterozygous males (*trpVa^1/+^* and *trpVa^2/+^*) attempted to copulate with females 6.3 ± 2.7 times (wild type), 7.7 ± 3.7 times (*trpVa^1/+^*) and 6.9 ± 2.7 times (*trpVa^2/+^*) in 5 min (Fig. 3*A*). In striking contrast, no *trpVa^1/2^* mutant males physically contacted the females in any of the trials (Fig. 3*A*).

To test the mating ability of *trpVa^1/2^* mutant males over a longer time frame, we grouped 5 males with 9-11 wild-type females for 3 d. The vast majority of females exposed to either wild-type or heterozygous males were fertilized (Fig. 3*B*). However, none of the wild-type females grouped with the *trpVa^1/2^* males were fertilized. The failure to fertilize females was not due to a defect in sperm production since the wild-type and mutant seminal vesicles contained similar numbers of sperm (Fig. 3*C*).

### Deafness Does Not Preclude Mating in Females.

Female receptivity to copulation affects mating success (22). To test whether loss of hearing impacts mating success of females, we grouped one wild-type male with 9-11 wild-type or *trpVa^1/2^* females for different durations and then scored the percentage of females that were fertilized by checking for viable progeny. After 5 min, 18.6 ± 19.5% of wild-type and 3.7 ± 7.4% of *trpVa^1/2^* females were fertilized (Fig. 3*D*). After grouping males and females for 30 min, 23.8 ± 15.9% of wild-type females were fertilized, compared to only 7.8 ± 10.7% of *trpVa^1/2^* females (Fig. 3*D*). After 60 min, 41.9 ± 24.6% of wild-type females were fertilized, while the number of *trpVa^1/2^* females that were fertilized went up to 22.9 ± 12.5% (Fig. 3*D*). While the differences between the wild-type and mutant females did not fall below the threshold for statistical significance (*P* < 0.05), there was a trend toward significance after 30 min (*P* = 0.053) and 60 min (*P* = 0.072). 60 min was required for a similar percentage of *trpVa^1/2^* females to be fertilized as after only 5 min for wild-type females. To test for longer durations we grouped 3 wild-type males with 7-11 wild-type or mutant females for 24 h and found that 71.6 ± 22.0% of wild-type and 57.1 ± 21.4% *trpVa^1/2^* females produced progeny–a difference that was not statistically significant (Fig. 3*E*). Thus, unlike with the mutant males, the *trpVa^1/2^* females still mate. However, the rate appears to be lower than with wild-type females, although the difference falls below the threshold for significance.

### Male Attraction to Sound Requires TRPVa.

**Fig. 4.**
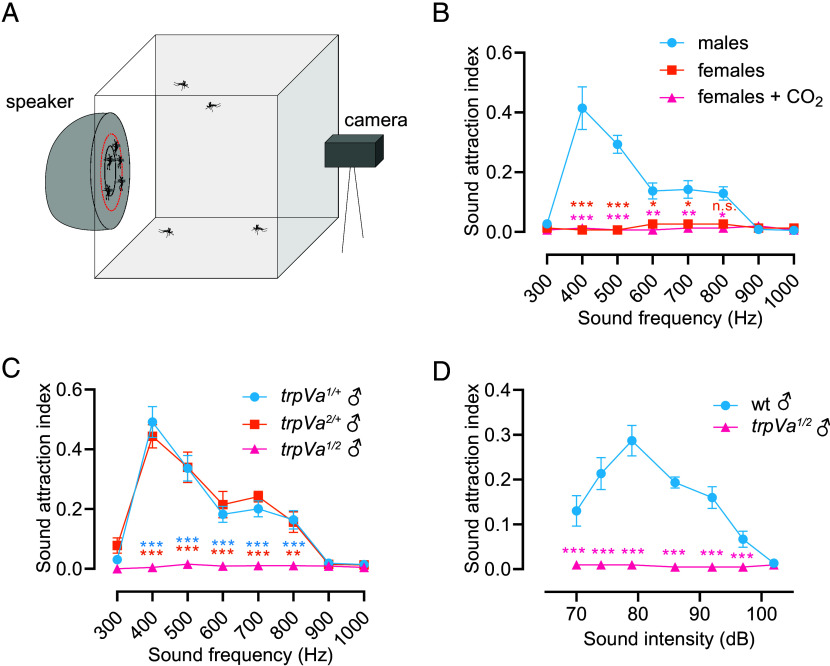
Attraction of male *Ae. aegypti* to sound requires *trpVa*. (*A*) Schematic of the setup for the sound attraction assay. Mosquitoes (10 to 30) were inserted in each 15 × 15 × 15 cm mosquito cage. A speaker was placed at a distance of 1 cm from the side of the cage opposite to the camera. Animals that landed in front of the speaker’s diaphragm (4.5 cm diameter) indicated by the red circumference were scored as attracted to the sound. The sound attraction index = N_2_/N_t_ (N_5_ = number of mosquitoes that landed within the tabulation zone for ≥2 s; N_t_ = total number of mosquitoes in the cage). (*B*) Attraction of wild-type male and female mosquitoes to various sound frequencies (Hz). For the CO_2_ application, we exposed females to 6 cycles of 20-s pulses of 5% CO_2_. n = 5-11 cages. (*C*) Sound attraction exhibited by *trpVa^1^*, *trpVa^2^*, and *trpVa^1/2^* males at different frequencies. n = 5-7 cages. (*D*) Impact of sound amplitude (dB) on attraction of males to 400 Hz, n = 5 cages. Two-way ANOVA with Tukey’s multiple comparison test for *B* and *C*. Two-way ANOVA with Sidak’s comparison for *D*. Means ± SEMs. n.s., not significant. *P < 0.05, **P < 0.01, and ***P < 0.001. Asterisks indicated in orange denote comparisons between males and females, whereas those in magenta denote comparisons between males and females + CO_2_ for *B*. Asterisks indicated in blue denote comparisons between *trpVa^1/+^* and *trpVa^1/2^*, whereas those in orange denote comparisons between *trpVa^2/+^* and *trpVa^1/2^* for *C*.

Male mosquitoes are attracted to female flight tones (44, 45), which vary in frequency depending on environmental conditions and the size of the mosquitoes (46, 47). We devised a sound-response assay to determine whether mutations in *trpVa* alter male behavioral responses to auditory stimuli at different frequencies. We applied 10-s sound stimuli (300 to 1,000 Hz pure sine tones) with a speaker on one side of a cage that contained 10 to 30 mosquitoes (Fig. 4*A*). We then scored the number of mosquitoes that landed and remained for ≥2 s inside a circular zone (4.5 cm diameter) at the center of the speaker during sound stimulation, and determined a sound attraction index (fraction of mosquitoes that landed in the circular zone out of the total number of mosquitoes in the cage). Under the temperature and humidity conditions used in this study (28 °C, 80% relative humidity), wild-type males were unresponsive to frequencies ≤300 Hz or ≥900 Hz, and were maximally attracted to frequencies between 400 and 500 Hz (Fig. 4*B* and Movie S3), which is near to the fundamental wingbeat frequency of females (46, 47). Female mosquitoes showed virtually no attraction to the sound stimuli (Fig. 4*B*). As females are stimulated by CO_2_, we tested whether they would show increased attraction to sounds in the presence of CO_2_. However, coapplication of sounds and CO_2_‚ elicited no attraction in females (Fig. 4*B*).

We then addressed whether loss of TRPVa in males impaired their sound-evoked attraction. The *trpVa^1/2^* mutant males showed no sound attraction regardless of the frequency, demonstrating that TRPVa is essential for sound attraction (Fig. 4*C* and Movie S4). This phenotype was recessive as the heterozygous *trpVa^1^*^/+^ and *trpVa^2^*^/+^ males displayed normal sound attraction (Fig. 4 *B* and *C*). To test whether the *trpVa^1/2^* mutants had reduced sensitivity to sounds, we compared the responses of wild-type and *trpVa^1/2^* males to the frequency that produced the strongest attraction (400 Hz) over a range of sound amplitudes from ambient levels (~70 dB) to loud sounds (102 dB). Wild-type males were attracted strongly to sounds between 74 and 86 dB and exhibited maximum attraction to 80 dB (Fig. 4*D*). The measured sound thresholds were high as the mosquitoes had to detect these tones at and above the 70 dB ambient background in the mosquito chamber. However, the *trpVa^1/2^* males were unresponsive to the 400 Hz tones at all amplitudes tested, further demonstrating the profound effect of the mutation on responding to auditory stimulation.

### *trpVa* Is Not Required for Flying, but for Initiating Flight That Is Induced by Sound.

**Fig. 5.**
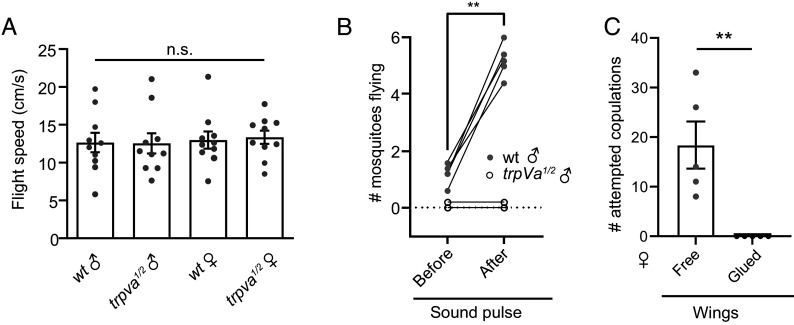
*trpVa* is critical for sound-induced flight initiation. (*A*) Flight speeds recorded for single wild-type and *trpVa^1/2^*males and females in a wind tunnel where airflow was applied in one direction at the rate of 0.5 m/s. (*B*) The number of wild-type and *trpVa^1/2^* male mosquitoes flying 1 s before and after a 500 ms, 400 Hz sound stimulus averaged over a total of 5 trials per cage. 10 to 15 males per cage, n = 3-5 cages. (*C*) Number of attempted copulations by wild-type males caged with a single tethered female that was either free or had glued wings. 10 to 15 males per cage, n = 5 cages. We used 15 × 15 × 15 cm cages in all cases. Kruskal–Wallis test with Dunn’s multiple comparisons for *A*. Mann–Whitney *U* test for *B* and *C*. Means ± SEMs. n.s., not significant. **P < 0.01.

In addition to chordotonal neurons, we found that *trpVa^QF2^*/+ driven expression of *QUAS-mCD8::GFP* labeled several axons in the VNC of males and females (*SI Appendix*, Fig. S4). Therefore, we tested whether the absence of sound attraction in *trpVa^1/2^* was due to a locomotor defect. Because mating in *Aedes* occurs during flight, we analyzed the flight speed of individual mosquitoes in a small wind tunnel (*SI Appendix*, Fig. S5). The flight speeds of *trpVa^1/2^* males and females were indistinguishable from wild-type mosquitoes (Fig. 5*A*). Although their ability to fly was unaffected, we observed that the *trpVa^1/2^* males were less inclined to take to flight when in a cage with wild-type females. While 6 out of 10 wild-type males could be seen in flight at any given time during 47 s of the recording, *trpVa* males did not take to flight at all (Movie S5). Previous work has revealed a flight initiation reaction of males in response to female flight tones (48). To test whether hearing was the critical cue that triggered flight, we applied a 400 Hz sound pulse for 500 ms (80 dB) to wild-type or *trpVa*-null males. Wild-type males that were resting on the cage walls or floor responded to the sound by initiating flight (Fig. 5*B* and Movie S6). In contrast, sound did not cause *trpVa* males to initiate flight (Movie S7).

To determine whether the sound of wingbeats is critical for triggering copulation, we tethered wild-type females and observed that their wingbeats almost instantaneously attracted males who immediately attempted copulation (Fig. 5*C* and Movie S8). Next, we tethered wild-type female mosquitoes that had their wings glued, thereby rendering them unable to generate sounds from wing beating. We released free-flying wild-type males into the enclosure and observed that the males did not locate the females and initiate copulation (Fig. 5*C* and Movie S9). Altogether, these observations indicate that loss of *trpVa* profoundly disrupts a critical step in mating-related behaviors, which is sound-induced flight initiation.

### Male Copulatory-Like Abdominal Bending Induced by Sound Requires *trpVa*.

**Fig. 6.**
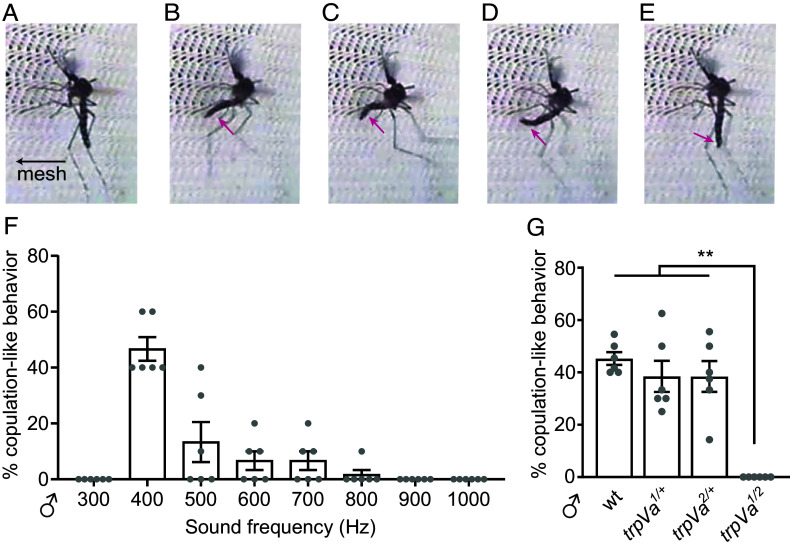
*trpVa^1/2^* males do not exhibit sound-induced copulatory-like behavior. (*A–E*) Images showing copulatory-like behavior by a wild-type male exposed to 400 Hz pure sine tone applied on the outside of the cage. (*A*) Male landing on the mesh near the speaker. (*B*) The male starts to bend its abdomen (arrow) in the direction of the speaker. (*C*) The abdomen (arrow) is bent further until it touches the mesh. (*D*) Abdomen (arrow) curves, emulating copulation. (*E*) The abdomen (arrow) returns back to its resting position. (*F*) Percentages of males that showed copulatory-like behavior induced by different frequencies. Each trial included 10 males per cage, n = 6 trials. (*G*) Percentages of males that showed copulatory-like behavior (≥3 abdomen bends) induced by 400 Hz during the 10 s sound period. Each trial included 6-12 males per cage, n = 6 trials. Kruskal–Wallis test with Dunn’s multiple comparison test for *G*. Means ± SEMs. n.s., not significant. * *P* < 0.05, **P < 0.01, and ***P < 0.001.

It was suggested decades ago that sound can induce copulatory-like behavior in male mosquitoes (7). In our experiments, males were most sensitive to 400 to 500 Hz sound. To determine whether sound-evoked copulatory behavior is strongest in response to a 400 to 500 Hz stimulus, we placed males in a cage and directed localized sound (300 to 1,000 Hz) toward a ~64 cm^2^ mesh area on one side of the cage. We found that during the 400 to 500 Hz sound stimulus, wild-type males landed on the cage mesh next to the sound source and started bending their abdomen repeatedly, exhibiting copulatory-like behavior (Fig. 6 *A*–*F* and Movie S10). This behavior was robust, as many wild-type and heterozygous *trpVa* males displayed this response within 1 s of starting the sound stimulus (Fig. 6*G*; wild type, 45.3 ± 5.9%; *trpVa^1^*/+, 44.7 ± 19.7%; *trpVa^2^*/+, 41.8 ± 14.5%). The males did not show copulatory-like behavior in response to 300 Hz sound and much lower responses when exposed to sound frequencies ≥500 Hz (Fig. 6*F*). In contrast to wild-type and *trpVa* heterozygous males, none of the *trpVa* transheterozygous males exhibited copulatory-like behavior to the applied sound (Fig. 6*G*).

### Establishing Fundamental Wingbeat Frequency Does Not Require Auditory Feedback.

**Fig. 7.**
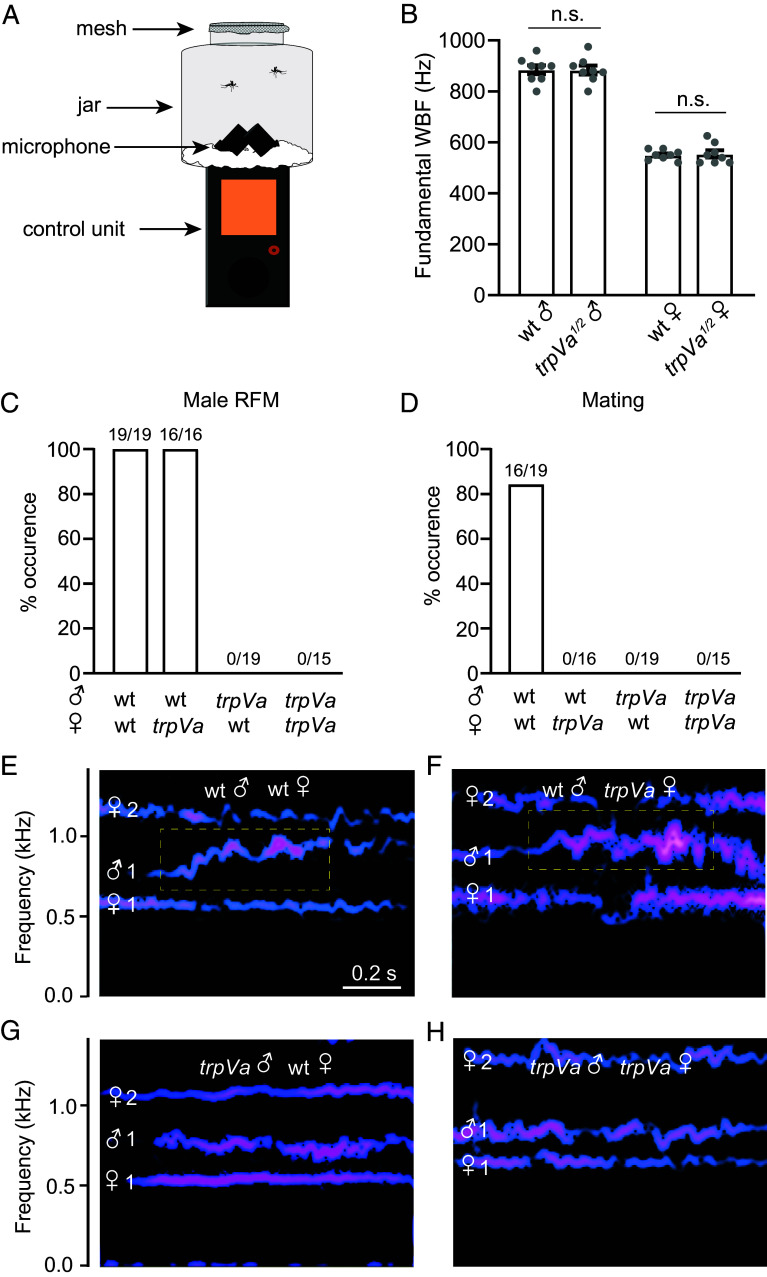
*trpVa* is required for RFM. (*A*) Schematic of experimental setup for measuring WBFs by a single male and female pair male in a 500 mL bottle jar. (*B*) Fundamental WBFs (Hz) of free-flying wild-type and *trpVa^1/2^* males and females, n = 8. (*C*) Percentage of RFM occurrence exhibited by a wild-type male or a *trpVa^1/2^* male when paired with either a wild-type female or a *trpVa^1/2^* female (we abbreviated *trpVa^1/2^* as *trpVa* for brevity). (*D*) Percent occurrence of mating exhibited by a wild-type male or a *trpVa^1/2^* male when paired with either a wild-type female or a *trpVa^1/2^* female. (*E–H*) Representative traces of the fundamental WBFs and harmonics (Hz) of one male and one female pair. The harmonics (1 and 2) are indicated. The dashed yellow boxes indicate when RFM occurred. (*E*) Wild-type male and female. (*F*) Wild-type male and *trpVa^1/2^* female. (*G*) *trpVa^1/2^* male and wild-type female. (*H*) *trpVa^1/2^* male and female. Mann–Whitney *U* tests. Means ± SEMs. n.s., not significant.

Male *Ae. aegypti* have higher fundamental WBFs than females (19). The question arises as to whether the fundamental WBFs of males and females depend on auditory feedback. To record flight tones, we released mosquitoes in a small enclosure (volume 500 mL), and recorded their wingbeats using a field microphone placed within the cage (Fig. 7*A*). Under our rearing conditions, the fundamental WBFs of 7- to 10-d-old mosquitoes were 885.0 ± 49.6 Hz for wild-type males, 883.8 ± 50.6 Hz for *trpVa^1/2^* males, 550.0 ± 19.8 Hz for wild-type females, and 554.4 ±19.8 Hz for *trpVa^1/2^* females (Fig. 7*B*). Thus, there were no significant differences between the wild-type and mutant males (*P* = 0.86) and the wild-type and mutant females (*P* = 0.82). These findings demonstrate that establishment of normal fundamental WBFs does not depend on auditory feedback.

### RFM Requires *trpVa*.

When the two sexes fly within close proximity, the males rapidly modulate their fundamental WBFs (22, 23, 49). In *Culex quinquefasciatus*, *Anopheles coluzzii*, and *Anopheles gambiae*, the sharp increases in the WBFs are followed by rapid oscillations at the higher frequencies, a phenomenon referred to RFM (23, 49). As shown in *C. quinquefasciatus*, the RFM in the male is followed by RFM in the female (23). In *Ae. aegypti*, the males also show RFM; however, the frequency modulation may not be as rapid as in *Culex* and *Anopheles*, and it is unclear whether the female displays RFM (22).

We took advantage of the *trpVa^1/2^* mutants to address whether RFM still occurs if one of the two insects is deaf. We monitored WBFs with free flying pairs consisting of one male and one female since tethered males do not exhibit RFM (23). In addition, using free flying pairs enables us to correlate RFM with mating. Consistent with previous work, we identified RFM as sharp changes in the male WBF (~1,250 Hz/s or ~100 Hz in 80 ms) followed by oscillations at the higher frequencies for ~150 ms to several seconds while both mosquitoes were in flight (23, 49). As reported previously (22), all wild-type males performed RFM when paired with wild-type females (19/19; Fig. 7 *C* and *E*). Similarly, we found all wild-type males displayed RFM in the presence of *trpVa^1/2^* females (16/16; Fig. 7 *C* and *F*). However, unlike *C. quinquefasciatus*, the female *Ae. aegypti* did not display RFM in any pairings with either wild-type males (n = 19) or *trpVa^1/2^* males (n = 19).

To determine whether *trpVa^1/2^* males exhibit RFM, we paired one mutant male with a wild-type female. We found that none of the *trpVa^1/2^* males performed RFM regardless of whether they were paired with a wild-type female (0/19; Fig. 7 *C* and *G*) or a *trpVa^1/2^* female (0/15; Fig. 7 *C* and *H*). Thus, we conclude that *trpVa* is required for RFM.

In many mosquitoes, such as *C. quinquefasciatus* (23), *An. coluzzii*, *An. gambiae* (49), and *Ae. aegypti* (22), mating is correlated with RFM. In support of these previous findings, in wild-type pairings, 100% of the matings were preceded by RFM (Fig. 7 *C* and *D*), although we occasionally observed RFM that did not result in mating within the 2-min observation period following RFM (3/19; 15.7%). As expected, *trpVa^1/2^* mutant males, which did not produce RFM, did not mate (Fig. 7*D*), consistent with the data presented above demonstrating that *trpVa^1/2^* mutant males do not mate (Fig. 3*B*). These data indicate that *trpVa* is required to perform RFM, and support the conclusion that RFM is a prerequisite for mating to occur in *Ae. aegypti*.

## Discussion

### Auditory Cues Are Required and Sufficient for Copulatory Behavior.

Prior to this work, multiple studies demonstrated that hearing the wing vibrations of the other sex influences mating in *Ae. aegypti* (7, 21, 22). Indeed, it is well known that males are attracted to the female wingbeat (7). Therefore, we expected mating success would be affected by eliminating the ability of the mosquitoes to hear. However, it was an open question as to the extent of the impact of deafness on mating by males or females. We found that deafness did not prevent *trpVa^1/2^* females from mating and producing progeny, although loss of *trpVa* increased the time needed for successful reproductive behavior.

Distinct from females, the deaf *trpVa^1/2^* mutant males did not mate. Wild-type males copulated with females multiple times within 5 min, whereas deaf *trpVa^1/2^* males did not mate successfully even after 3 d. This contrasts starkly from deaf male *Drosophila*, which still mate since they employ multiple modes of communication as part of their courtship and mating behavior (2). Due to the brief, seconds-long mating in flight, the strong reliance on detection of wing beating by *Ae. aegypti* would be far more time efficient than the multisensory courtship ritual display by fruit flies, which mate for many minutes on the ground. Nevertheless, despite our findings that deaf males do not mate, these results do not exclude one or more other cues contribute to male mating success, such as visual stimuli.

### JO Neurons Required for Sound Detection.

It has been known for many years that in the JO of *Ae. aegypti*, there are four classes of neurons (A-D), while females are endowed with just three classes (A-C) (31, 35). The precise roles of the various classes have not been defined in *Aedes*. In *Drosophila*, there are five classes of JO neurons (A-E). The JO-A and JO-B neurons sense sound-induced vibrations of the arista, while the JO-C and JO-E detect wind and gravity (27–29, 36–39). The role of the JO-D neurons in *Drosophila* is less clear. In *Ae. aegypti*, we detected expression of the *trpVa* reporter in all four classes of JO neurons in males, but only in the JO-A and JO-B neurons in females. Since mutation of *trpVa* causes loss of sound detection in both males and females, we propose that the JO-A and JO-B neurons are required for sound detection in *Ae. aegypti*, as is the case in *Drosophila*.

Using the *Aedes trpVa* reporter, we found that the axonal projections of the JO neurons into the auditory brain center (the AMMC) are sexually dimorphic. The more dispersed pattern in the male AMMC is not likely to be due simply to the presence of a fourth class of JO neuron (JO-D) in males. Rather, it appears that the projection patterns of the JO-A and JO-B neurons are divergent in the male and female AMMC, which might account in part for sexually dimorphic behavioral responses to sound.

### Distinct Requirements for Hearing for RFM and for Establishing Fundamental WBFs.

The fundamental WBFs of males and females are distinct (21). However, it should be noted that the WBFs we determined are not identical to other studies since they are impacted by temperature and humidity, as well as the age and the size of the mosquitoes (46, 47). Nevertheless of significance here, we found that the fundamental WBFs of deaf *trpVa^1/2^* mutant males and females were indistinguishable from wild-type mosquitoes. Thus, we conclude that auditory feedback is not necessary for producing normal fundamental WBFs in *Ae. aegypti*. Consistent with our finding that hearing does not alter the fundamental WBF, we found that a wild-type male mosquito still performs RFM when paired with a deaf *trpVa* female. However, deaf *trpVa* males do not perform RFM. Since they cannot detect the wingbeats of the female conspecific, this indicates that the ability to hear the female wing beating is essential to motivate the RFM. Consistent with previous reports (22, 23, 49), production of RFM in *Aedes* males is an outstanding predictor of mating intention since all mating events were preceded by RFM, and there were relatively few instances of RFM that did not result in mating.

Unlike *An. gambiae* and *C. quinquefasciatus*, where mating is initiated by females detecting and entering male swarms (50, 51), in *Ae. Aegypti,* mating is initiated by males approaching females and performing RFM (22). Our findings support these conclusions since we show that *Ae. aegypti* males perform RFM and females do not. Additionally, deaf *trpVa^1/2^*males do not perform RFM and do not mate. It has been proposed that the onset of RFM facilitates the ability of a male to control its position during flight so that the male can grab the female for copulation (22, 49). Then, RFM might continue to facilitate stable flight during copulation (22, 49). However, in the case of *Ae. aegypti*, the females may not need to make the same sort of flight adjustments afforded by RFM for successful mating.

### Future Perspective.

The demonstration that *trpVa* is essential for mating success has potential implications for the sterile insect technique (SIT), which involves release of large numbers of sterile males (52). Once sterile males mate with wild-type females, the females become refractory to subsequent mating, thereby preventing the females from being fertilized and producing progeny. However, the efficacy of SIT in suppressing populations of *Ae. aegypti* is limited in part by an insufficient level of mating competitiveness by the sterile males (53, 54). The demonstration that *trpVa* is required for male mating may provide the conceptual basis for enhancing the mating competitiveness of male mosquitoes, by increasing the excitability of *trpVa*-expressing neurons, which in turn might elevate male sensitivity to the female wing-beating.

## Materials and Methods

### Mosquito Rearing and Maintenance.

*Ae. aegypti* Gainesville mosquitoes were used as the wild-type strain (Benzon Research, Carlisle, PA). Mosquitoes were reared at 28 °C, 80% relative humidity under 14 h light:10 h dark cycles. Larvae were hatched and kept in reverse osmosis filtered water and fed small fish food granules (TetraMin Tropical Granules, #16122, Tetra Co., Melle, Germany). Adults were fed 10% sucrose ad libitum and blood-fed using an artificial membrane feeding system (Hemotek, ltd., Blackburn, UK) with warmed, defibrillated sheep’s blood (HemoStat Laboratories, DSB250). The mosquito rearing and maintenance procedures were performed in an ACL-2 facility, which was approved by the UCSB Institutional Biosafety Committee. Pupae were sex-separated manually after visual inspection based on size. The sexes were confirmed as adults 12 h posteclosion. Because we did not retain pupae in cases in which we were unsure of the sex based on size, we successfully separated males and females nearly 100% of the time. Nevertheless, we reared only 10 to 20 mosquitoes per cage, so that if we did have a contaminant we would simply not use mosquitoes from that cage. Adult males are easily distinguishable from females with the naked eye as the males have bushy antennae, longer maxillary palps, and are generally smaller in size. Prior to each experiment, we checked cages to ensure that sex separation was successful. The *trpVa* mutants were outcrossed to the wild-type Gainesville background for ≥5 generations.

### Relatedness of *Aedes* TRPVa Protein with Similar Proteins from Other Species.

The following protein sequences were downloaded from the NCBI protein database: *Ae. aegypti* TRPVa (AAEL020482-PA, XP_021695000.1), *An. gambiae* (AGAP000413-PA, XP_310685.5), *D. melanogaster* Iav (CG4536-PA, NP_572353.1), *Homo sapiens* TRPV6 (NP_061116.5, NM_018646.6), and *Mus musculus* TRPV6 (NP_071858.3, NM_022413.4). The protein sequence alignments and guide tree were analyzed using Molecular Evolutionary Genetic Analysis (MEGA) X software (https://www.megasoftware.net). The six TMDs in TRPVa were predicted by the TMpred online analysis program (https://bio.tools/TMPred).

### Generation of *trpVa* Mutants.

The three *trpVa* alleles (Fig. 1*A*) were generated by CRISPR-mediated homology-directed repair (HDR) using the Gainesville *Ae. aegypti* strain. Each *trpVa* allele included a gene encoding either *DsRed* (*trpVa^1^* and *trpVa^QF2^*) or *GFP* (*trpVa^2^*) expressed under control of the *3xP3* promoter to allow identification of transgenic mosquitoes on the basis of eye fluorescence. The insertions in *trpVa^1^* and *trpVa^QF2^* interrupted the DNA region coding for residue 206. The insertion in *trpVa^QF2^* disrupted the codon for residue 579.

To construct the *trpVa* alleles, we first generated the p*AaU6-LgRNA-3xP3-DsRed* and p*AaU6-LgRNA-3xP3-GFP* plasmids (*SI Appendix*, Fig. S6 *A* and *B*) (55) by In-Fusion assembly (Takara Bio, Shiga, Japan. # 638948). These plasmids contained the *Ae. aegypti U6* promoter (*AAEL017774*), a modified gRNA scaffold (55), and either the *3xP3-DsRed-SV40* or the *3xP3-eGFP-SV40* fragment. The *U6* promoter and gRNA scaffold fragments were synthesized by gBlocks™ Gene Fragments (IDT). The gRNA was expressed under control of the *U6* promoter. The *3xP3-eGFP* and *3xP3-DsRed* fragments were PCR-amplified from p*3xP3-UASpBacFPN* (DGRC #1287) and p*HD-DsRed* (Addgene #51434).

The *trpVa^1^*allele was generated using p*AaU6-LgRNA-3xP3-DsRed* with a gRNA (5′-TGTCCCAGCGCGCCATCGGA-3′) that targeted exon 3 of *trpVa*. ~1.3 kb upstream and ~1.0 kb downstream homology arms on either side of the Cas9 cut site in exon 3 were generated by PCR amplification of the designated regions from genomic DNA extracted from the *ubiL40-Cas9* strain (56). The upstream homology arm was amplified with the following primers and inserted into the PacI site of p*AaU6-LgRNA-3xP3-DsRed*: 5′-GTGGTGCAATTTGATTCCGTACA-3′ and 5′-GATGGCGCGCTGGGACACGT-3′. The downstream homology arm was amplified using the following primers and inserted into the NheI site of p*AaU6-LgRNA-3xP3-DsRed*: 5′-GGACGGTTCTTCCTTCCACGGGAT-3′, and 5′-TGCAAATATCCCTTGTGCTC-3′. The plasmid clone (p*AaU6*-*trpVa^1^*-*LgRNA-arm1-3x P3-DsRed-arm2*) was verified by DNA sequencing, amplified using an endotoxin-free midi-prep kit (ZYMO Research, #D4200) and eluted in nuclease-free water (Ambion, #AM9939). ~400 ng/μL of p*AaU6*-*trpVa^1^*-*LgRNA-arm1-3xP3-DsRed-arm2* was injected into ~1,000 *ubiL40-Cas9* embryos (56) using a quartz needle (quartz glass capillaries; OD, 1.0 mm; ID,0.7 mm; length, 10 cm; Sutter Instrument, cat. # Q100-70-10, pulled by a micropipette puller, Sutter Instrument, p-2000). 52 surviving G_0_ mosquitoes were collected; the males and females were separated and then introduced into two 17.5 × 17.5 × 17.5 cm cages (BugDorm-4S1515). The G_0_ females were crossed to ~10 wild-type males, and the G_0_ males were crossed to threefold the number of wild-type mosquitoes. The progeny (G_1_) were screened for eye fluorescence (3xP3-DsRed expression). >20 DsRed-positive G_1_ larvae were identified, and a strain without the Cas9 marker was verified by PCR using the following primers: forward 5′-CCGGTCAGTGCTCATGTACGAT-3′, and reverse 5′-TCTCGAACTCGTGGCCGTTC-3′.

The *trpVa^2^*allele was generated using a gRNA (5′-TACAGGAAGTAGAACGCCT-3′) targeting exon 5 of *trpVa*. ~1.6 kb upstream and ~1.9 kb downstream homology arms on either side of the Cas9 cut site in exon 5 were generated by PCR amplification of the designated regions from genomic DNA extracted from the Gainesville strain. The upstream homology arm was amplified with the following primers and inserted into the PacI site of p*AaU6-LgRNA-3xP3-GFP*: 5′-TCTCCGCATGATCCTTTGGG-3′ and 5′-CCTGGGAAAATCCGAACAGAA-3′. The downstream homology arm was amplified with the following primers and inserted into the NheI site of p*AaU6-LgRNA-3xP3-GFP*: 5′- CGTTCTACTTCCTGTACAAAG 3′, and 5′-TCGACACTAGCCACTGCCACCTTCATCTC-3′. The clone (p*AaU6*-*trpVa^2^*-**L*gRNA-arm1-3xP3-GFP-arm2*) was verified by DNA sequencing, amplified using an endotoxin-free midi-prep kit (ZYMO Research, #D4200) and eluted in nuclease-free water (Ambion, #AM9939). A mixture of 300 ng/μL Cas9 protein (PNA Bio), 400 ng/μL U6-gRNA plasmid, and 400 ng/μL dsDNA plasmid donor was injected into ~2,000 wild-type Gainesville embryos. ~100 surviving G_0_ mosquitoes were crossed to the Gainesville strain and the positive progeny were screened for the presence of the GFP marker. 8 GFP-positive G_1_ larvae were isolated. We verified the *trpVa^2^* mutation by PCR with the following primers: forward 5′-TCTACGGTGGAAGTACATGGAT-3′, and reverse 5′-TGAACTTCAGGGTCAGCTTGC-3′.

The *trpVa^QF2^* allele was generated using the same gRNA and arms used to create *trpVa^1^*. A fragment containing *T2A::QF2-SV40* was PCR-amplified from p*AC-DsRed-QF2* (gift from C. Potter, Johns Hopkins School of Medicine, Baltimore, MD) (33) by adding sequences encoding T2A in the forward primer. *T2A::QF2* was inserted in-frame after the upstream arm. The clone (p*AaU6*-*trpVa^QF2^*-*LgRNA-arm1-T2A::QF2-3xP3-DsRed-arm2*) was verified by DNA sequencing, amplified using an endotoxin-free midi-prep kit (ZYMO Research, #D4200) and eluted in nuclease-free water (Ambion, #AM9939). ~400 ng/μL p*AaU6*-*trpVa^QF2^*-*LgRNA-arm1-T2A::QF2-3xP3-DsRed-arm2* was injected to ~2,000 *ubiL40-Cas9* embryos (56). ~100 surviving G_0_ mosquitoes were individually crossed to wild-type mosquitoes of the opposite sex, and the progeny were screened for eye fluorescence on the basis of the DsRed marker. >20 DsRed-positive G_1_s were identified and a strain without the Cas9 marker (*opie-DsRed*) was verified by PCR using the following primers: forward 5′-CCGGTCAGTGCTCATGTACGAT-3′, and reverse 5′-ACGCGCGACATTTTCAAACA-3′.

### Generation of the *Ae. aegypti QUAS-mCD8::GFP* Reporter.

To generate transgenic mosquitoes expressing *15x-QUAS-mCD8::GFP*, we introduced the transgene in the *white* gene on the first chromosome (56), which enabled us to select homozygous animals by screening for mosquitoes with ECFP fluorescence eyes and white eyes. To create the plasmid for injections, we first used In-Fusion assembly to construct p*AaU6-white-gRNA-arm1-15xQUAS-3xP3-ECFP-arm2*, which consisted of the following fragments: p*AaU6-white-gRNA-arm1-arm2*, which was PCR amplified from p*AaU6-white-gRNA-arm1-arm* (gift from O. Akbari, UCSD, San Diego, CA) (56), and *15xQUAS-SV40* and *3xP3-ECFP*, which was PCR-amplified from p*BAC-ECFP-15xQUAS-SV40* plasmid (gift from C. Potter) (57). We then PCR amplified *mCD8::GFP* DNA from pUAST-*mCD8::GFP* (Addgene #17746) and introduced it into the XhoI site of p*AaU6-white-gRNA-arm1-15xQUAS-3xP3-ECFP-arm2* to create p*AaU6-white-gRNA-arm1-15xQUAS-mCD8::GFP-3xP3-ECFP-arm2* (shortened name: p*Aa-white-15xQUAS-mCD8::GFP*; *SI Appendix*, Fig. S6*C*).

We injected 400 ng/μL p*Aa-white-15xQUAS-mCD8::GFP* into ~1,000 *ubiL40-Cas9* embryos. ~100 surviving G_0_ mosquitoes were individually crossed to wild-type mosquitoes of the opposite sex, and >20 *ECFP-*positive G_1_ larvae were isolated. During outcrossing, we removed the *Cas9* transgene by selecting against *opie2-DsRed* expression in the gut. We verified the *QUAS-mCD8::GFP* transgene by PCR using the following primers: forward 5′-TGGGACTCAGTGAGGAGGAC-3′ and reverse 5′-AAAGGCATTCCACCACTGCT-3′).

### Conditions and Ages of Mosquitoes for Behavioral Assays.

We carried out all behavioral assays at 28 °C and 80% relative humidity in 15 × 15 × 15 cm cages, unless stated otherwise. Despite the small size of these cages, the males were able to exhibit looping or zig-zag flight patterns, which are associated with mating behavior (42), while the mosquitoes were in flight [Movie S1 (male looping), and Movie S2 (mating)]. All mosquitoes used were sex-separated by size beginning from the pupal stage. We used 7- to 10-d-old adult mosquitoes, unless stated otherwise. All transfers of mosquitoes from one cage to another were performed with a mouth aspirator that consisted of a sterile filter attached to a mouthpiece at one end and a broken-off 10 mL pipette at the other end. In order to give the mosquitoes time to recover from the transfer, we performed experiments 24 h after transferring them to the cages used for the behavioral assays.

### Sound Source and Measurements for Sound Assays.

All of our sound attraction and electrophysiology assays employed Creative Pebble V2 speakers (Creative Technology Ltd., Singapore) due to their relatively small dimensions and because they generated pure sine tones that matched the high fidelity of a professional grade studio monitor (Yamaha HS8; *SI Appendix*, Fig. S7). Sound intensity measurements were performed with a sound meter (Digisense 20250-29) using a C-weighted scale (Fig. 4*D*) or with a Tascam DR-44WL (TEAC Corporation, Tokyo, Japan) audio recorder and analyzed in Audacity which generated amplitude measurements in dBFS (*SI Appendix*, Fig. S8).

### Sound Attraction Assays.

The sound attraction assays were performed in 15 × 15 × 15 cm mesh cages. To prevent introduction of human odor, all of the following manipulations were performed while wearing nitrile gloves. We placed the speaker 1 cm from the side of the cage. The sound stimulus was introduced as a pure sine tone for 10 s using tone generator software (Audacity) at 78 dB. To test for possible sound distortion in the cage, we measured sounds at different locations inside an empty cage and found that the amplitude of the applied sound tones was maximum at the side of the cage nearest to the speaker (*SI Appendix*, Fig. S8). For each trial, 10 to 30 mosquitoes were introduced into the cage. In some experiments with female mosquitoes, we placed a CO_2_ tube outlet just above the speaker on the outside of the cage and exposed the mosquitoes to six pulses (20 s each) of 5% CO_2_ separated by 30 s intervals.

To assay sound attraction, we monitored the mosquitoes using an HD web camera (Logitech C920s) at 30 frames/s. The diaphragm of the speaker is ~3.5 cm in diameter with casing accounting for the rest of the body of the speaker. When attracted to the sounds emanating from the speakers, the mosquitoes landed on the mesh directly in front of the diaphragm, gathering in a cluster that spread from the center of the diaphragm to ~4.5 cm outward. Therefore, we counted the number of mosquitoes that landed on the mesh within a circle (4.5 cm diameter) centered around the middle of the speaker for ≥2 s during the duration of the sound stimulus (N_2_). N_t_ = total number of mosquitoes in the cage. Sound attraction index = N_2_/N_t._ The mosquitoes that were present in the circle zone before the sound was applied (N_0_) were not counted in N_2_ or N_t_. To increase mosquito activity and to decrease random landings in the circle zone, the cage was gently shaken once before initiating the sound stimulus.

### Flight Assay.

To assay flight speed, we constructed a small wind-tunnel assay system (*SI Appendix*, Fig. S5). The wind-tunnel consisted of a transparent plastic tube 4.2 cm in inner diameter and 15 cm in length. One end of the tube was covered with mesh and acted as a barrier preventing the mosquitoes from flying out of the tube, while allowing air to be blown into the tube at a constant rate. Constant airflow was created using a fan-blade mounted on a 3 to 6 V DC motor controlled by an Arduino UNO R3. Mosquitoes flew for the longest periods of time along the length of the tube when airflow velocity was 0.5 m/s at the center of the tube, as measured by a thermal anemometer (HT9829, Hti-Xintai). The opposite end of the tube was docked to a 15 × 15 × 15 cm cage by inserting the wind-tunnel tube through the entry port of the cage and wrapping the cage’s mesh around the opening so that the only outlet for the mosquitoes from the cage was the tunnel. Cages contained ~10 mosquitoes and the mosquitoes were allowed to freely enter the wind tunnel on their own. All experiments were performed at 28 °C and 80% humidity. Mosquito flight speed was assessed only in cases in which an individual mosquito flew into the tunnel. If more than one mosquito entered the tunnel, those recordings were discarded. Mosquito flight inside the tunnel was recorded using a camera (Logitech C920s) at 30 frames/s and flight trajectories of individual mosquitoes were tracked using FlyTracker (Caltech). After recording each mosquito, the cages were changed to avoid recording the same mosquito. The average flight speeds of each mosquito were calculated from a planar projection of the flight trajectories plotted on the length-wise (x) and height-wise (y) axes of the tube as *v_total_* = √(*v*_x_^2^ + *v*_y_^2^) and summarized using GraphPad Prism.

### Sound-Evoked Flight Initiation.

We introduced 7- to 10-d-old wild-type or *trpVa^1/2^* males into 15 × 15 × 15 cm cages. We allowed the mosquitoes to adapt to these cages for ~24 h before the start of the assay. A speaker was placed on the top of the cage and 500 ms long, 400 Hz sine tones were applied for a total of 5 times with a 2-min interval between each trial. The average number of mosquitoes over all 5 trials that were in flight for 1 s before and 1 s after the sound pulse were compared. Movies were recorded with a Logitech C920s camera at 30 frames/sec.

### Sound-Evoked Field Recordings.

7- to 10-d-old male and non-blood-fed female mosquitoes were immobilized on microscope slides with beeswax. The sound stimuli were produced using tone generator software (Audacity), and delivered to the mosquitoes by placing a speaker (Creative Pebble V2) at a distance of 7 cm from the mosquito. Two glass electrodes (thin-wall glass capillaries; OD, 1.0 mm; ID, 0.75 mm, length, 76 mm; World Precision Instruments, cat. #TW100F-3) were prepared with a micropipette puller (Sutter Instrument, P-97). The electrodes were filled with Ringer’s solution (2 mM CaCl_2_, 140 mM NaCl, 5 mM KCl, 1 mM MgCl_2_, and 10 mM HEPES pH 7.4). The tips of the electrodes and the surface of the mosquito thorax were covered with electrode cream (Parker, cat. #17-05). The reference electrode was placed on the surface of the thorax. The recording electrode was placed on the ventrolateral portion of the JO. Pure sine tones ranging from 300 to 1,000 Hz at 83 dB at the position of the JO were played for 50 ms. Sound-induced signals were amplified using an IE-210 amplifier (Warner Instruments). The data were acquired at 2 kHz. The gain factor was 50×, and the input impedance of the amplifier was 10^11^ Ω. The data were digitized with a PowerLab 4/30 device and LabChart 6 software (AD Instruments). The electrode resistance was 1.8 ± 0.2 MΩ as measured using an Axon 200B amplifier and a Digidata 1440A (Molecular devices). The amplifier gain was accounted for in the measurement of the final amplitude of the field potential. Normalized data (*SI Appendix*, Fig. S3 *E* and *F*) were calculated using the maximum response for each animal.

### Copulatory-Like Behavioral Assay Induced by Sound.

The assay was modified from the sound attraction assay described above. For each experiment, 6-12 virgin males were introduced into a 15 × 15 × 15 cm mesh cage. A 10 s, 400 Hz sound at 78 dB was played from the speaker as described above. A male was scored as exhibiting copulatory-like behavior if it landed on the mesh and showed abdominal bending behavior (≥3 times) during the 10 s sound period. The percentage of males that showed copulatory-like behavior per cage trial was calculated as N_c_/N_total_ × 100, where N_c_ is the number of males showing copulatory behavior and N_total_ is the total number of males in the cage.

### Attempted Copulation Assays.

One male was grouped with 10 virgin wild-type females in a 15 × 15 × 15 cm cage and video-recorded using a camera (Logitech C920s). A copulation attempt was scored when the male approached a female and made physical contact (58). The number of attempts were scored during a 5 min period.

### Long-Term Male Mating Assays.

For the long-term male mating assay, 5 males were grouped with 9-11 virgin wild-type females in a 15 × 15 × 15 cm cage. After 3 d, the males were removed and the females were blood-fed. Individual females were aspirated into individual tubes with damp egg collection papers for egg laying 4 d after blood-feeding. Females were released from their individual tubes and reverse osmosis water was added to each tube to hatch the eggs laid 4 d after the females were added to the tubes. The percentages of female mosquitoes that produced progeny per trial were calculated.

### Sperm Quantification.

To quantify mature sperm, we dissected tissue containing seminal vesicles and accessory glands from 4- to 9-d-old male mosquitoes into 50 μL of PBS using fine forceps. The sperm were dissociated and mixed to a uniform concentration by repeated pipetting using a P200 tip. 10 μL of the cell suspension was added to a hemocytometer, and the cells were counted at 200× magnification on a Zeiss LSM 700 microscope with the sample illuminated with a yellow halogen light source (HAL100; *SI Appendix*, Fig. S9). We found that if we performed the imaging with a white light source, we were unable to detect unstained sperm if the aperture was open wide enough for Köhler illumination. However, if we constricted the field aperture, this enhanced the contrast sufficiently to visualize the sperm. For each sample, we calculated the average sperm per 100 nL and then multiplied by 500 (50 μL/0.1 μL) to obtain total sperm counts. Due to the thinness of the sperm relative to the depth of the solution under the coverslip of the hemocytometer, the sperm were not all in the same focal plane. Therefore, to count the sperm, we manually changed the focus while scanning over the grid on the hemocytometer.

### Short-Term Female Mating Assay.

To perform a short-term assay for successful insemination of females, we grouped one wild-type male with 9-11 virgin females in a 15 × 15 × 15 cm cage for 5, 30, or 60 min. After each trial, the males were removed and females were blood-fed 1 d later. Eggs were collected and hatched from individual females in tubes using the method described above. The percentages of females with offspring were scored.

### Long-Term Female Mating Assay.

For the long-term female mating assay, 3 wild-type males were grouped with 9-11 virgin females in a 15 × 15 × 15 cm cage for 24 h. Then, the males were removed, and the females were blood-fed 1 d later. The eggs of each female were collected and hatched individually by transferring single females to egg-collecting tubes, as described above. The percentages of females with offspring were scored.

### Measuring RFM.

One male and one female (7 to 10 d old) were aspirated into a sterile, empty plastic 500 mL bottle (GenClone 25-228, Genesee Scientific). The bottle was closed at the top with a cheese-cloth mesh held in place with a rubber band. Mosquitoes were allowed to fly freely inside the bottle. Wingbeats were recorded with a Tascam DR-44WL audio recorder inserted into the bottle through a perforation made in the bottom (Fig. 7*A*). The signals were analyzed using Audacity software. The spectrogram traces were generated using Audacity after Hann windowing. We followed the definition of RFM as described (22), which is the fundamental wingbeat frequency changing by ≥100 Hz in ≤80 ms (1,250 Hz/s) (22).

### Immunostaining of Cryosections of JO.

*Ae. aegypti* JO neurons are covered with a black cuticle, rendering them difficult to image after performing immunostaining on whole mounts of antennae. Therefore, to perform staining, we sectioned *trpVa^QF2^*/*QUAS-mCD8::GFP* antennae. We anesthetized 7- to 10-d-old mosquitoes on ice, removed the heads with a razor blade, and placed them in 1.5 mL Eppendorf tubes with 4% paraformaldehyde in PBST [PBS (137 mM NaCl, 2.7 mM KCl, 8 mM Na_2_HPO_4_, and 2 mM KH_2_PO_4_) + 0.3% Triton X-100] for 1 h on ice. We washed the heads 3 times for 15 min in PBST, added 10% sucrose diluted in 0.3% PBS, rotated for 1 h at room temperature, and then incubated the heads overnight at 4 °C in 30% sucrose. We then placed the heads in molds (Fisher Brand #22363552) with OCT (Tissue-Tek® O.C.T. compound, Sakura), froze the heads on dry ice, and obtained sections using a Leica Cryostat CM1850. The antennal sections were collected on SuperFrost Plus slides (Fisher Scientific), rinsed 3 times for 15 min in 0.3% PBST, and then incubated for 2 d at 4 °C with primary antibodies in blocking solution (Alexa 488 conjugated CD8 alpha, Invitrogen, #MCD0820,1:500 and Alexa 633 conjugated phalloidin, Invitrogen, #A22284,1:1,000). The slides were rinsed 3 times for 15 min in 0.3% PBST, and mounted in Vectashield (Vector Laboratory, H-1000). We performed staining with anti-mCD8 rather than anti-GFP since this latter reagent would have also detected the 3xP3-ECFP marker used for identifying *QUAS-mCD8::GFP* transgenic animals. We used phalloidin to label F-actin, which is highly enriched in scolopidia. The images were collected using a Zeiss LSM 700 confocal laser scanning microscope with either a Zeiss Plan-Apochromat 20×/0.8 objective or a Plan-Neofluar 40×/1.3 oil objective.

### Immunostaining of Brains and Ventral Nerve Cord.

Brains or ventral nerve cords were dissected from 7- to 10-d-old mosquitoes anesthetized on ice, and fixed with 4% paraformaldehyde in PBST for 1 h on ice. The tissues were washed 3 times for 15 min in PBST and blocked with 10% normal goat serum (MP Biomedicals) in PBST for 30 min at room temperature. The samples were then incubated with the primary antibodies (rabbit anti-CD8 alpha, #EPR21769, 1:200 and mouse anti-nc82, Developmental Studies Hybridoma Bank, 1:50) in blocking buffer (10% goat serum in PBST) overnight at 4 °C, washed 3 times for 15 min in PBST, and incubated with secondary antibodies (goat anti-rabbit Alexa Fluor 488, cat. #A48282, Invitrogen 1:1,000; goat anti-mouse Alexa Fluor 568, cat. #A11004, Invitrogen 1:1,000) in blocking buffer overnight at 4 °C in the dark. The tissues were washed 3 times for 15 min in PBST and mounted using VECTASHIELD (Vector Laboratory, H-1000). The images were acquired using a Zeiss LSM 700 (brains) and LSM 900 (VNCs) confocal laser scanning microscope and a Zeiss Plan-Apochromat 20×/0.8 objective.

### Statistical Analyses.

All statistical analyses were performed using GraphPad Prism software. Because all of our data were non-normal, we used nonparametric tests. We used the Mann–Whitney *U* test to test statistical significance between two groups and the Kruskal–Wallis test with Dunn’s multiple comparison test for multiple groups. We conducted two-way ANOVA with Tukey’s or Sidak’s multiple comparison when appropriate. All error bars represent means ± SEMs. *P < 0.05, **P < 0.01, and ***P < 0.001.

## Supplementary Material

Appendix 01 (PDF)

Movie S1. Looping behavior of mosquitoes in a 15 x 15 x 15 cm cage. The cages contained 3 male and 3 female wild-type mosquitoes. The movies were acquired at 30 frames/sec, and played at 15 frames/sec. Shown are the tracks of the mosquitoes in flight over the course of 20 frames, before dissolving and starting new tracks. Looping flight showing figures of 8 can be seen, including one at the end of the movie. The mosquitoes were visually assessed for body size, abdomen shape, and antennal morphology to determine and annotate male (M) and female (F) mosquitoes.

Movie S2. Mating behavior of mosquitoes in a 15 x 15 x 15 cm cage. The cages contained 3 male and 3 female wild-type mosquitoes. The movies were acquired at 30 frames/sec, and played at 15 frames/sec. Shown are the tracks of the mosquitoes in flight over the course of 20 frames, before dissolving and starting new tracks. Mating behavior, represented by mid-flight coupling of a male and female, occurs towards the end of the movie. The mosquitoes were visually assessed for body size, abdomen shape, and antennal morphology to determine and annotate male (M) and female (F) mosquitoes.

Movie S3. Sound attraction assay with wild-type males. In each experiment 10-30 wild-type males were inserted in a 15 x 15 x 15 cm mosquito cage. A 10-sec 400 Hz pure sine tone was applied using a speaker placed outside of the cage. Males were strongly attracted to the speaker area when the sound was turned on.

Movie S4. Sound attraction assay with *trpVa^1/2^* males. In each experiment 10-30 *trpVa^1/2^* males were inserted in a 15 x 15 x 15 cm mosquito cage. A 10 sec 400 Hz pure sine tone was applied using a speaker placed outside of the cage. Males showed no attraction to the speaker area when the sound was turned on.

Movie S5. *trpVa^1/2^* males are less inclined to fly. 10 free-flying wild-type (left) or *trpVa^1/2^* (right) male mosquitoes were housed with 10 wild-type females.

Movie S6. Wild-type male flight initiation in response to a 400 Hz acoustic stimulus. In each experiment 10-15 wild-type males were exposed to a 400 Hz (80 dB) pure sine tone for 500 ms. The number of mosquitoes flying 1 second before and after the stimulus was recorded.

Movie S7. *trpVa^1/2^* male flight initiation in response to a 400 Hz acoustic stimulus. In each experiment 10-15 *trpVa^1/2^* males were exposed to a 400 Hz (80 dB) pure sine tone for 500 ms. The number of mosquitoes flying 1 second before and after the stimulus was recorded.

Movie S8. Males attempted copulations with a tethered female with free wings. 10 wild-type males were released in a cage containing a single tethered female and the number of attempted copulations was recorded over the course of 5 minutes.

Movie S9. Males did not attempt to copulate with a tethered female with glued wings. 10 wild-type males were released in a cage containing a single tethered female that had wings glued together using UV-polymerized Bondic glue. The movie shows a 1.22 minutes-long clip out of a movie that was recorded for 5 minutes.

Movie S10. Copulation-like behavior in response to sound. 10 wild-type males were exposed to a 400 Hz pure sine tone (83 dB) for 10 seconds. 4 males landed on the mesh near the speaker and started to bend their abdomens, extending the tip of their abdomen towards the speaker. This is similar to the behavior exhibited by a male copulating with a female.

## Data Availability

All study data are included in the article and/or supporting information. All videos, audio files and electrophysiology used to generate the data in this study are available at Dryad (DOI: 10.5061/dryad.qz612jmrb) (59).
