# Supplementary material for: Deafness due to loss of a TRPV channel eliminates mating behavior in Aedes aegypti males
Source: Proc Natl Acad Sci U S A. 2024 Nov 4;121(47):e2404324121. doi: 10.1073/pnas.2404324121 (PMC11588044; doi:10.1073/pnas.2404324121)
Supplement: Supplementary file 1 — Appendix 01 (PDF) [file pnas.2404324121.sapp.pdf]

PNAS

Supporting Information for
Deafness due to loss of a TRPV channel eliminates mating behavior in *Aedes aegypti* males

Yijin Wang*, Dhananjay Thakur*,**, Emma Duge*, Caroline Murphy, Ivan Girling, Nicolas A. DeBeaubien, Jieyan Chen, Benjamin H. Nguyen, Adishthi S. Gurav, and Craig Montell**

*These authors contributed equally.

**Corresponding authors: Dhananjay Thakur and Craig Montell
Email: dthakur@ucsb.edu and cmontell@ucsb.edu

This PDF file includes:

Figures S1 to S9
Legends for Movies S1 to S10

A

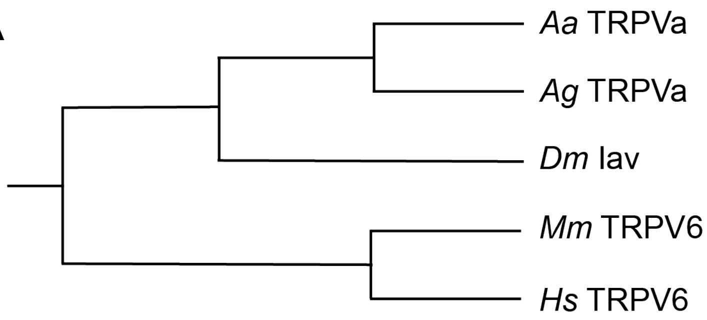

B

	Aa	*Ag*	*Dm*	*Mm*	*Hs*
Aa	100.0				
Ag	75.8	100.0			
Dm	74.8	75.1	100.0		
Mm	28.8	28.5	26.8	100.0	
Hs	28.5	28.2	27.0	88.8	100.0

Fig. S1. Comparison of *Ae. aegypti* TRPVa to the most related proteins in selected animals. (*A*) Phylogenetic tree showing relatedness of *Ae. aegypti* TRPVa (*Aa* TRPVa; *AAEL020482*, XP 021695000.1), *Anopheles gambiae* TRPVa (*Ag* TRPVa; *AGAP000413*, XP310685.5), *Drosophila melanogaster* Iav (*Dm*_Iav; NP572353.1), *Mus musculus* TPRV6 (*Mm* TRPV6; NP_071858.3), and *Homo sapiens* TRPV6 (*Hs* TRPV6; NP_061116.5). (*B*) Percent amino acid identities of TRPV proteins indicated in (*A*) with *Aa* TRPVa.

```
Aa TRPVa   MVQFDWRRMCRKKRKVPQGGAILDQV----ISQSANASNQCLLYKMANYKRGGDLIDAFQVGGQKAVEQLIREQFGVFMYNNGRGQIINRAEYLRWKYMD   96
Ag TRPVa   MVHLDPLRLCRKKRKLPQGGAILDQV----ISQSASASNQCLLYKMANYKRGGDLIDAFQIGGQKAVEQLIREQFGVFMYNKGRGQIINRAEYLRWKYMD   96
Dm Iav     -MKFLLKKCLRKKAPEMKPGAILDAV----ISQSSATACKCLLYKLADYKRGGDLIDAINSGLIAVEQLIREQFGVFMYNDGKGQVINRAEFLRWKYRD   95

Aa TRPVa   NHEVVIPIEASLSPHDPLGKWVDHKACWQMQYRGLLGESLLHVLIICDTKIHTKLARILLRVFPELSIDVMEGEEYLGASALHLAIAYSNNELVGDLIDA   196
Ag TRPVa   NHEVIIPIEASLSPHDPLSKWDDHKACWQMQYRGLLGESLLHVLIICDTKVHTKLARILLRVFPEQSIDVMEGEEYLGASALHLAIAYSNNELVGDLIDA   196
Dm Iav     HTEVTIPIEASLSIHDPLGKWEDHKACWQMQYRGALGESLLHVLIICDSKVHTKLARVLLRVFPNLALDVMEGEEYLGASALHLSIAYSNNELVADLIEA   195

       trpVa1 and trpVaQF2
Aa TRPVa   GADVSQRAIGRFFLPRDQQGLKPAKNTDYEGLAYLGEYPLAWAACCANESVYNLLLECGADPNAQDSFGNMILHMVVV-------CDKLDMFG-YALRHP   288
Ag TRPVa   GADVSQRATGRFFLPRDQQGLRPAKTTDYEGLAYLGEYPLAWAACCANESVYNLLLECGADPNAQDSFGNMILHMVVV-------CDKLDMFG-YALRHP   288
Dm Iav     GADIHQRAIGSFFLPRDQQRANPAKSTDYEGLAYMGEYPLAWAACCANESVYNLIVDCGSDPDAQDSFGNMILHMVVV-------CDKLDMFG-YALRHP   287

Aa TRPVa   KLPCKNGIVNESGLTPLTLACRLGRDEVFREMLELSAREFWRYSNITCSGYPLNALDTLMPDGTTNWNSALFIILNGTKEEHLNMLDGGIVERLLDEKWK   388
Ag TRPVa   KLPCKNGIVNAAGLTPLTLACRLGRDEVFREMLELSAREFWRYSNITCSGYPLNALDTLMPDGSTNWNSALFIILNGTKEEHLNMLDGGIVERLLDEKWK   388
Dm Iav     KTPAKNGIVNQTGLTPLTLACKLGRAEVFREMLELSAREFWRYSNITCSGYPLNALDTILPDGRTNWNSALFIILNGTKPEHLDMLDGGIIQRLLEEKWK   387

                          TM1                                                          TM2
Aa TRPVa   TFARNQFIKRLLILALHLFCLSCSVYLRPVRVFKEESEEGSDGGD-----GVGTAAPDGVDD-DEDIDLTTWFRYGFEIATVMGVLSYVVLQQGDEIKNQ   482
Ag TRPVa   TFARNQFIKRLLILAIHLFCLSCSVYLRPVRV--FADDEGEEGGDATDDDGADDGGPMAVDAVDDDIDLTTWVRYGFEVATVMGVLSYVVLQQGDEIKNQ   486
Dm Iav     TFAQNQFLKRLLILSTHLLCLSVSVYLRPAHD-GEAEDEDSEGSDASAAALLDIQSDEGDSG-GGLYNAQTVARYCAEFATLVGVLSYVIFQQGDEIKNQ   485

                            TM3                             TM4                    TM5    trpVa2
Aa TRPVa   GLISFI-KSLGNAPAKAIFLISNLMILACIPFRMMGDVEHEEAILLFAVPGSWFLLMFFAGAIGLTGPFVTMIFSMITGDMFTFGIIYMIVLFGFSQAFY   581
Ag TRPVa   GFFSFL-KSLSQAPAKAIFLISNILILACIPLRMMGDTETEEAILLFAVPGSWFLLMFFAGAIGLTGPFVTMIFSMITGDMFTFGIIYMIVLFGFSQAFY   585
Dm Iav     GLSAFL-KQLSHAPAKAIFLFSNLLILACIPFRLIGDTDTEEA LIFAVPGSWFLLMFFAGAIRLTGPFVTMIYSMITGDMFTFGIIYCIVLCGFSQAFY   584

                                                      TM6
Aa TRPVa   FLYKGHPEAEDSPFGSYFGTWMGLFQTTLGDYDYADLNLTTYPNLAKTVFIIFMIFVPILLLNMLIAMMGNTYAYVIEQAEKEGMKQWAKIVVNLERAVK   681
Ag TRPVa   FLYKGHPNAEDSPFGSYFGTWMALFQTTLGDYDYADLNLTTYPNLAKTVFVIFMIFVPILLLNMLIAMMGNTYAYVIEQAEKEGMKQWAKIVVNLERAVT   685
Dm Iav     FLYKGHPQVQSTMFNTYTSTWMALFQTTLGDYNYPDLNQTTYPNLSKTVFVIFMIFVPILLLNMLIAMMGNTYVTVIEQSEKEWMKQWAKIVVTLERAVP   684

Aa TRPVa   QEDAKKYLEEYSIGLGPSDDPRYEIRGVMVIKSKSKTRARQRKGAVSNWKSVLRVTLNELKKRNMTGEELRRIMWGRSSITSPAKISKKKKIYEEE----   777
Ag TRPVa   QDDAKRYLEEYSIGLGPSDDPRYETRGVMVIKSKSKTRARQRKGAVSNWKSVLRVTLNELKKRSMTGEELRRIMWGRSSITSPAKIAKKKRPGEVDDLLG   785
Dm Iav     QADAKGYLEAYSIPLGPSDDSGFEVRGVMVIKSKSKTRAKQRKGAVSNWKRVGRVTLTALKKRGMTGEEMRRLMWGRASISSPVKVTKQK--------LK   776

Aa TRPVa   DPFAITNAIDVMSFTQDIVMVSSEPTGPPTIDP-------------SKPATIAVKPQTAPAAQPIPEIPPQPPLVKRQSVVSAPPA--YDDFPPMKDYKD   862
Ag TRPVa   DPFAITAAIDVMSFTQDIVMVSTDTVCPITIPPAAAPGTAAPKAGGTKPVVVAPPP---PAAATAVQQPPPPATTRRHSTASAPATMGLGVEAALPGYKD   882
Dm Iav     DPYNLHTDSDFTN-AMDMLTFASNPASSNGV---------------TLRSVTAPPP-----------APPAP---------------------------D   822

Aa TRPVa   PLRELVIISE--SPSVDDHYVQSCKTLANDASTLDHVHEINLTKQNQLRQEE---------QKQ-----QQQQT---------------LGQL-NPFLEA   930
Ag TRPVa   PLRELVIISE--SASVDENYAQNVKTLAIDASTLDHVHEIDISQQQQQQQQQ---------QQQQPQQPQQQQN---------------LGQL-TLFQNP   955
Dm Iav     PFRELIMMSDQRPETHDPHYFAGLQQLANKA--FDLVEQTMKTQPQAPVAKKVDPLPVASVAKASPAAPATQATATAAAASDLMAMPLPISNLSNLFQDP   920

Aa TRPVa   KDVVDPVKEREFLKTLEALEDTDSEAAEKPVLGKISLIRRAKSAVSRTTSRKKKT-------DQHPLFTIAWDDKNLTRTHPEDF--GALNTAYEYSAED   1021
A  TRPVa   KDVVDPVREREFLKTLEALEDTDSEAGEKPVLGKISLIRRAKSAVSRSTSRKRKT-------DQHPLFMIAWEDKGDQQRHSTALYDGATGAAPSVGAP-   1047
Dm Iav     KDIVDPKKLEEMAMLAEVETEESDSGG-PILGKLSLAKRTHNALSKAEIKRDQQGFEGHSHGQFQPMSSVWAPPGLDVDTGFHFDEAVAEEVLTIEQE-   1018

Aa TRPVa   FKQDVDEEAREEEEDEGVTVEEVHRRMEELHHRGRASLERDSNSTES----------SKKHPHRK-------RQGMGRGRNNKVSPDTSNESVSG-----   1099
Ag TRPVa   -----GELGQPEQPEDAVTVEELHRRMEQFHQRASVR-ERDANSSESGSGKQHAGPKARKPSHHRDGGGSHHHHGLGRGKHNKISPDNSNESTGGGGGGG   1141
Dm Iav     AEVETEDGNGGQDSEDIPTAEEVHATMKQFHLRKCQPAQDEA---------------ARRAKSAR------------VRRRNKVSPEQSDDP--------   1083

Aa TRPVa   ------KKDKRMKSAP---TGGGGVRHRQLMDQ---------ETGGTSPPDPLEPWSTRDICNINKLLDTDTQDE   1156
Ag TRPVa   ASGHGRKQDKRMKSAPILGSGGAGSAGSSAGGRTVAPGGARPDDGTGSPPDPLEPWSTKNIMNINKLLDQDTTEE   1216
Dm Iav     --------DERSQ------------RGRSAYTR-----------RTQSPPDPLEPWSTRELQDINKILARK----   1123
```

Fig. S2. Sequence alignment of *Ae. aegypti* (*Aa*) TRPVa with *Anopheles gambiae* (*Ag*) TRPVa and *Drosophila melanogaster* (*Dm*) Iav. Amino acid identities among all three TRPV channels are in dark blue and identities among two of the three are in light blue. The running tallies of amino acids are shown to the right. The regions corresponding to the transmembrane domains (TM1 to TM6) are indicated by the orange lines, and the positions of the insertions in the $trpVa^{1}$, $trpVa^{2}$ and $trpVa^{QF2}$ alleles are indicated by the red arrowheads.

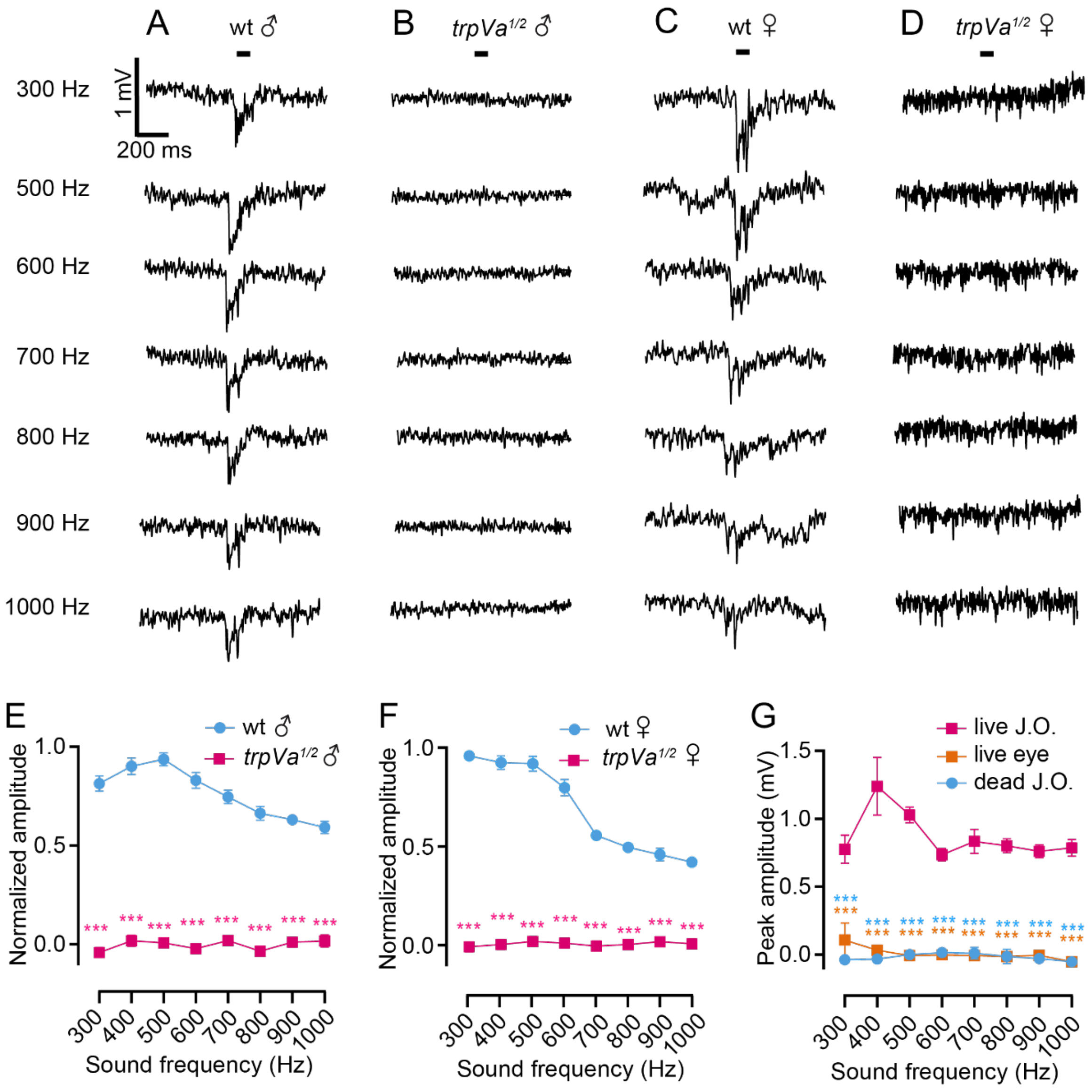

Fig. S3. *trpVa* is required for sound-evoked responses from the Johnston's organ (JO). (*A*–*D*) Traces showing sound-evoked extracellular potentials recorded from the JO in response to sound stimulation at different frequencies. The stimuli were 50 ms (black bars). (*A*) Wild-type male. (*B*) $trpVa^{1/2}$ mutant male. (*C*) Wild-type female. (*D*) $trpVa^{1/2}$ female. (*E*) Amplitudes of sound-evoked extracellular potentials normalized to the maximum response for each animal for the data shown in Figure 1*F*. (*F*) Amplitudes of sound-evoked extracellular potentials normalized to the maximum response for each animal for the data shown in Figure 1*G*. (*G*) JO potentials using an electrode placed on the JO of a live wild-type male, and a dead wild-type male to account for a possible artifact generated by the electrode or resonant vibrations induced in the dead mosquito. We also placed an electrode on the eye of a live wild-type male to examine whether a startle response from a mosquito generated an electrical artifact. Shown are average responses to stimuli from 300–1000 Hz at 83 dB. n = 3. Means ± S.E.M.s. Two-way ANOVA with Sidak's multiple comparison for *E* and *F*. Two-way ANOVA with Tukey's multiple comparison test for *G*. ***$P <$ 0.001. In *G*, the orange asterisks denote comparisons between responses from the eye and JO of live wild-type male mosquitoes. The blue asterisks denote comparisons between JO responses from dead and live wild-type male mosquitoes.

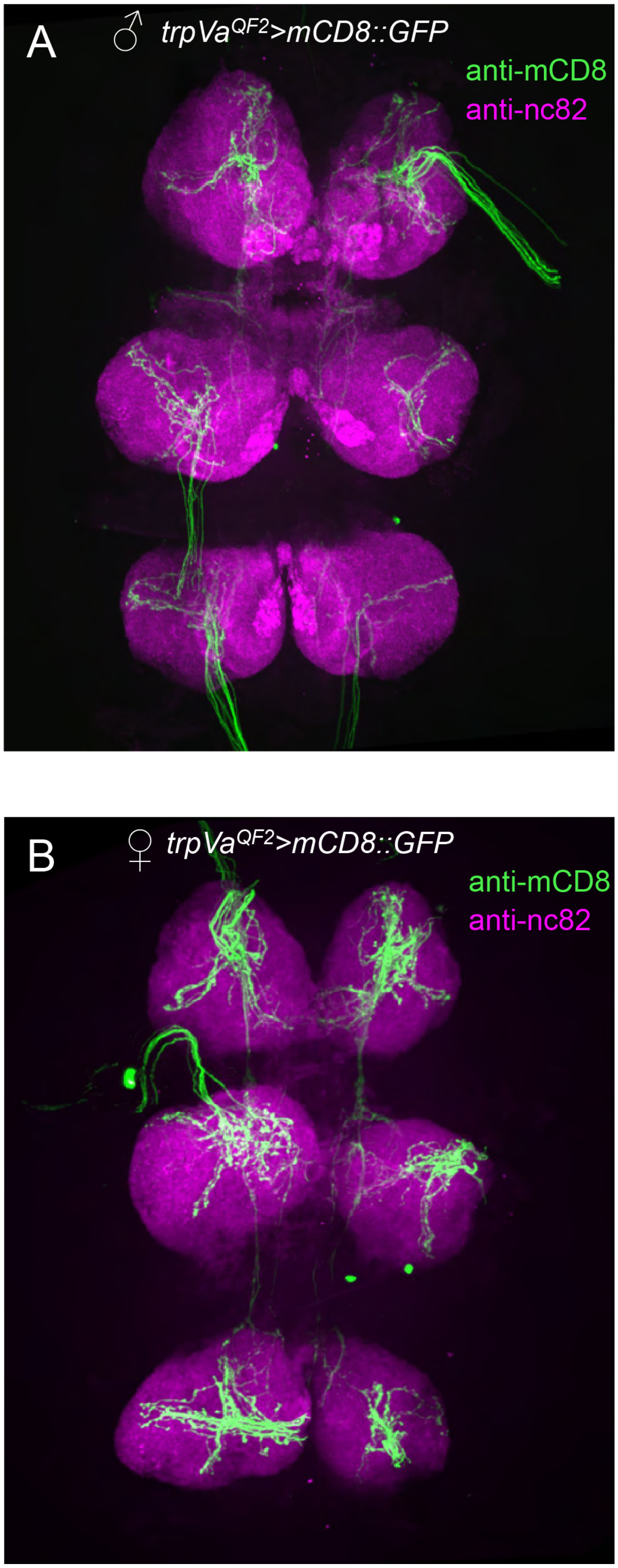

Fig. S4. Expression of the *trpVa* reporter (*trpVa*^{QF2}>*QUAS-mCD8::GFP*) in the ventral nerve cord. (*A*) Male. (*B*) Female. Anti-mCD8 (green) stains the *trpVa GFP* reporter and anti-nc82 (magenta) stains neuropils.

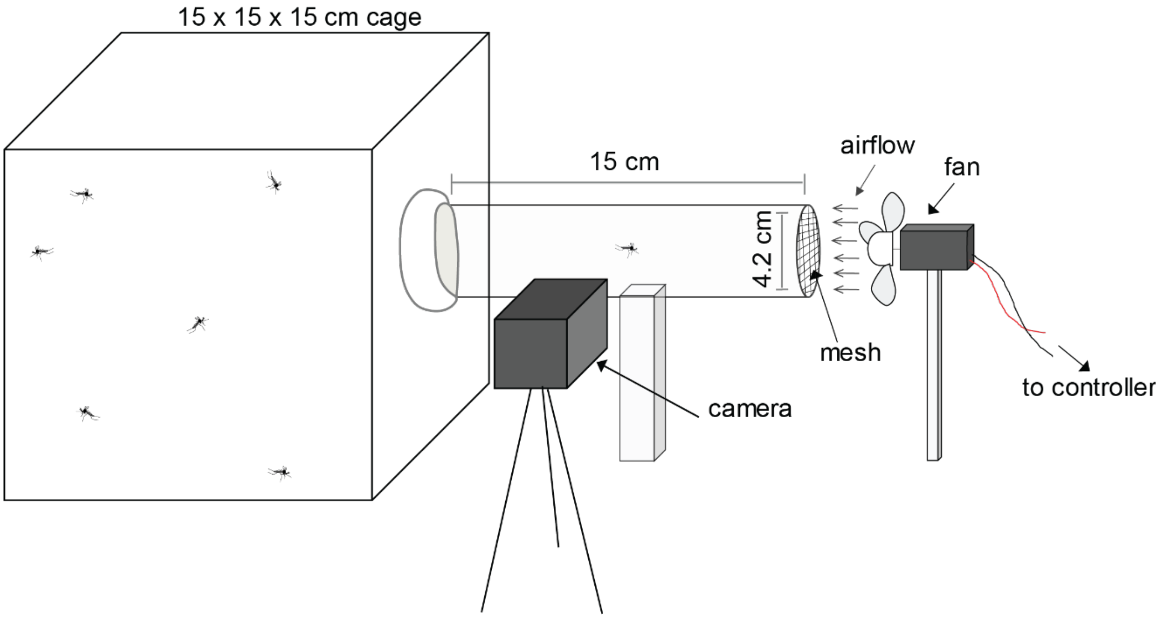

Fig. S5. Cartoon showing the 15 cm x 4.2 cm wind-tunnel assembly used to test flight speeds. A cage containing ~10 mosquitoes was attached to the open end of the tube. Individual mosquitoes were video-recorded when entering the tube from the cage on their own accord and flying along the length of the tube.

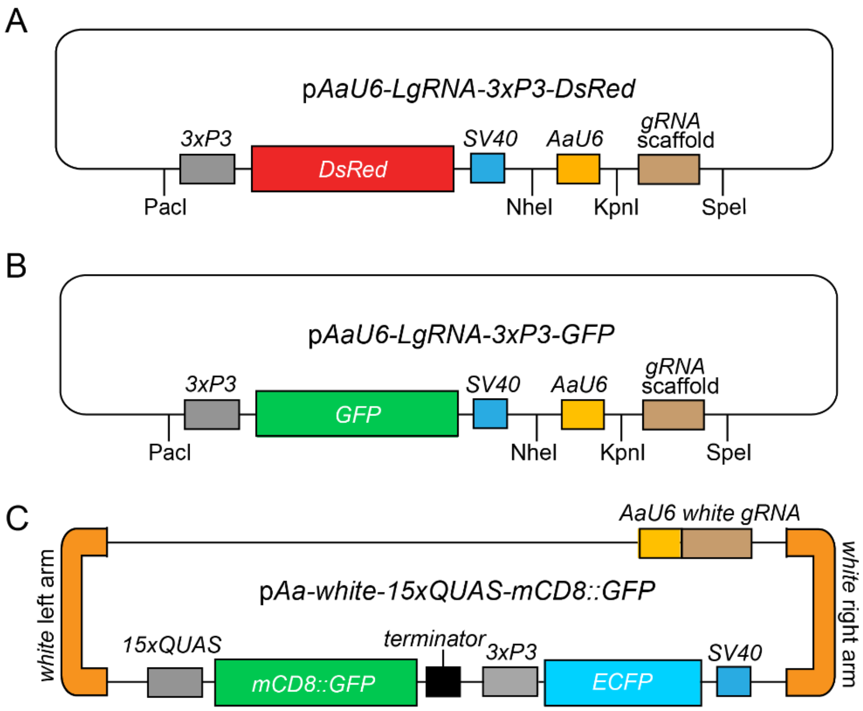

Fig. S6. Schematic of the plasmid constructs used to create the *trpVa* mutants and transgenic mosquitoes. (*A*) p*AaU6-LgRNA-3xP3-DsRed* plasmid used for engineering the knock-in construct for CRISPR-Cas9-mediated genome editing of *trpVa* with a *DsRed* marker. (*B*) p*AaU6-LgRNA-3xP3-GFP* plasmid used for engineering the knock-in constructs for CRISPR-Cas9-mediated genome editing of *trpVa* with a *GFP* marker. The PacI and NheI restriction sites were used to introduce the 5' and 3' homologous arms. The KpnI and SpeI restriction sites were used to insert the gRNAs. (*C*) p*AaU6-white-15xQUAS-mCD8-GFP* plasmid used for knocking in *15xQUAS-mCD8-GFP* into the *white* gene.

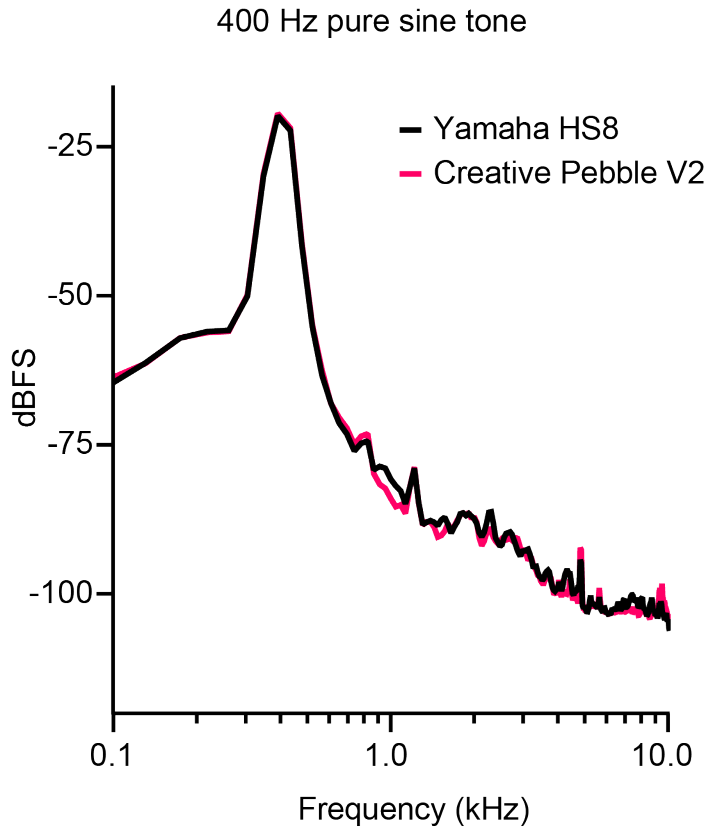

Fig. S7. Calibration of the speakers that were used for the behavioral and electrophysiology assays. A pure 400 Hz sine-tone was applied for 5 seconds through either a Yamaha HS8 studio monitor or a Creative Pebble V2 speaker. The sound amplitude was adjusted to be the same for both speakers when recorded using a TASCAM field microphone placed at the same distance from both speakers. Recorded sounds were analyzed using Audacity.

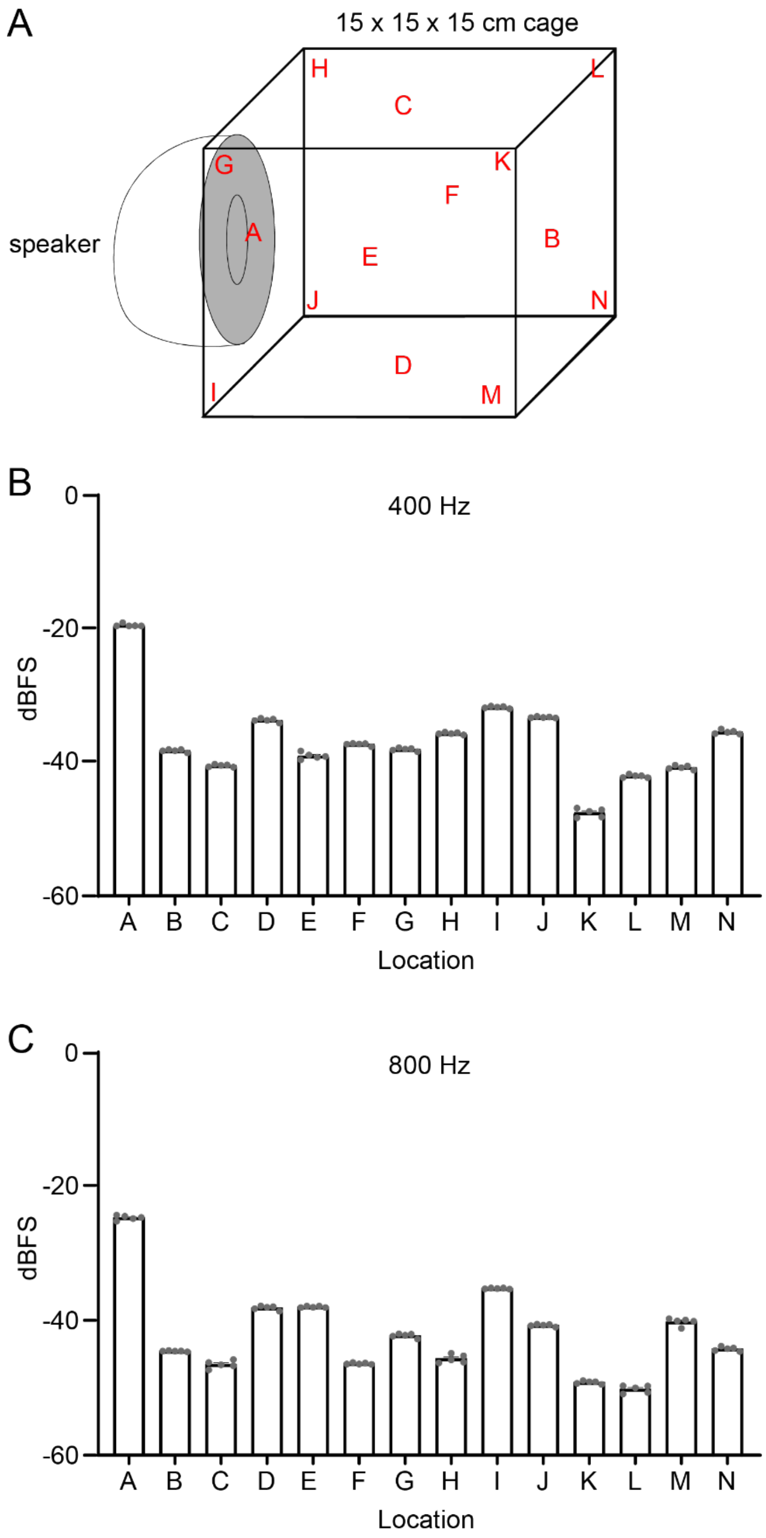

Fig. S8. Measurements of sound quality in the behavior test cage. (*A*) Cartoon showing the locations used for recording the 400 Hz and 800 Hz pure sine tones at 78 dB in a 15 x 15 x 15 cm cage that we used for the behavioral assays. (*B*) Responses at different locations to 400 Hz sounds. (*C*) Responses at different locations to 800 Hz sounds. There were no significant distortions of the sound amplitude at the different locations for either the 400 Hz or 800 Hz tones.

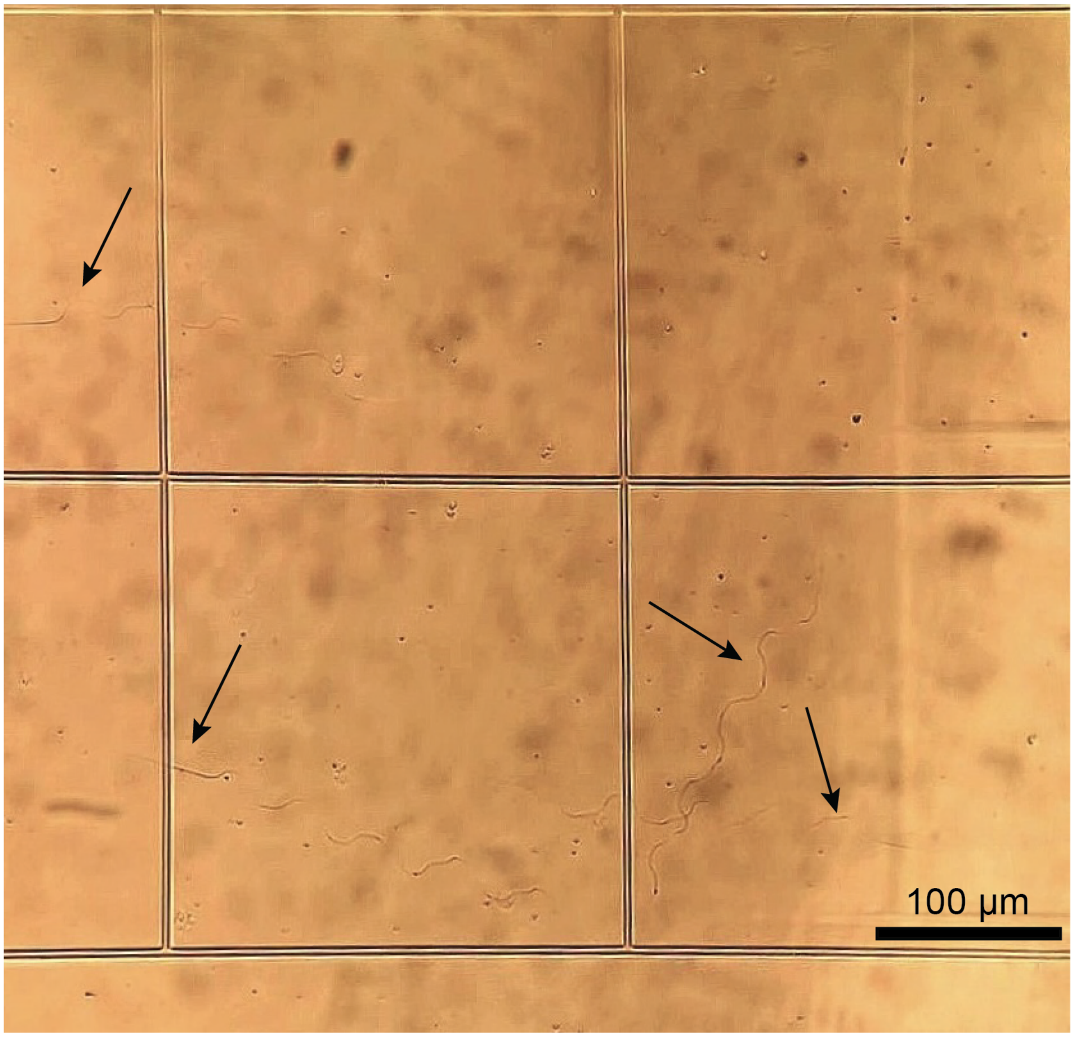

Fig. S9. Brightfield images of unstained male *Ae. aegypti* sperm on a hemocytometer. Sperm were counted using a Zeiss LSM 700 confocal microscope under a broadband yellow light from a halogen light source. The image was shot through the eyepiece using an iPhone 14 Pro. Sperm are indicated with arrows. Scale bar, 100 µm.

Move legends

Movie S1 (separate file). Looping behavior of mosquitoes in a 15 x 15 x 15 cm cage. The cages contained 3 male and 3 female wild-type mosquitoes. The movies were acquired at 30 frames/sec, and played at 15 frames/sec. Shown are the tracks of the mosquitoes in flight over the course of 20 frames, before dissolving and starting new tracks. Looping flight showing figures of 8 can be seen, including one at the end of the movie. The mosquitoes were visually assessed for body size, abdomen shape, and antennal morphology to determine and annotate male (M) and female (F) mosquitoes.

Movie S2 (separate file). Mating behavior of mosquitoes in a 15 x 15 x 15 cm cage. The cages contained 3 male and 3 female wild-type mosquitoes. The movies were acquired at 30 frames/sec, and played at 15 frames/sec. Shown are the tracks of the mosquitoes in flight over the course of 20 frames, before dissolving and starting new tracks. Mating behavior, represented by mid-flight coupling of a male and female, occurs towards the end of the movie. The mosquitoes were visually assessed for body size, abdomen shape, and antennal morphology to determine and annotate male (M) and female (F) mosquitoes.

Movie S3 (separate file). Sound attraction assay with wild-type males. In each experiment 10-30 wild-type males were inserted in a 15 x 15 x 15 cm mosquito cage. A 10-sec 400 Hz pure sine tone was applied using a speaker placed outside of the cage. Males were strongly attracted to the speaker area when the sound was turned on.

Movie S4 (separate file). Sound attraction assay with $trpVa^{1/2}$ males. In each experiment 10-30 $trpVa^{1/2}$ males were inserted in a 15 x 15 x 15 cm mosquito cage. A 10 sec 400 Hz pure sine tone was applied using a speaker placed outside of the cage. Males showed no attraction to the speaker area when the sound was turned on.

Movie S5 (separate file). $trpVa^{1/2}$ males are less inclined to fly. 10 free-flying wild-type (left) or $trpVa^{1/2}$ (right) male mosquitoes were housed with 10 wild-type females.

Movie S6 (separate file). Wild-type male flight initiation in response to a 400 Hz acoustic stimulus. In each experiment 10-15 wild-type males were exposed to a 400 Hz (80 dB) pure sine tone for 500 ms. The number of mosquitoes flying 1 second before and after the stimulus was recorded.

Movie S7 (separate file). $trpVa^{1/2}$ male flight initiation in response to a 400 Hz acoustic stimulus. In each experiment 10-15 $trpVa^{1/2}$ males were exposed to a 400 Hz (80 dB) pure sine tone for 500 ms. The number of mosquitoes flying 1 second before and after the stimulus was recorded.

Movie S8 (separate file). Males attempted copulations with a tethered female with free wings. 10 wild-type males were released in a cage containing a single tethered female and the number of attempted copulations was recorded over the course of 5 minutes.

Movie S9 (separate file). Males did not attempt to copulate with a tethered female with glued wings. 10 wild-type males were released in a cage containing a single tethered female that had wings glued together using UV-polymerized Bondic glue. The movie shows a 1.22 minutes-long clip out of a movie that was recorded for 5 minutes.

Movie S10 (separate file). Copulation-like behavior in response to sound. 10 wild-type males were exposed to a 400 Hz pure sine tone (83 dB) for 10 seconds. 4 males landed on the mesh near the speaker and started to bend their abdomens, extending the tip of their abdomen towards the speaker. This is similar to the behavior exhibited by a male copulating with a female.
